# Rational Design of Biocompatible Ir(III) Photosensitizer to Overcome Drug‐Resistant Cancer via Oxidative Autophagy Inhibition

**DOI:** 10.1002/advs.202407236

**Published:** 2024-11-14

**Authors:** Mingyu Park, Jung Seung Nam, Taehyun Kim, Gwangsu Yoon, Seoyoon Kim, Chaiheon Lee, Chae Gyu Lee, Sungjin Park, Kochan S. Bejoymohandas, Jihyeon Yang, Yoon Hee Kwon, Yoo Jin Lee, Jeong Kon Seo, Duyoung Min, Taiho Park, Tae‐Hyuk Kwon

**Affiliations:** ^1^ Department of Chemistry Ulsan National Institute of Science and Technology (UNIST) Ulsan 44919 Republic of Korea; ^2^ X‐dynamic Research Center Ulsan National Institute of Science and Technology (UNIST) Ulsan 44919 Republic of Korea; ^3^ Institute for Cancer Genetics Department of Genetics and Development Columbia University Medical Center New York NY 10032 USA; ^4^ Herbert Irving Comprehensive Cancer Center Columbia University Irving Medical Center New York NY 10032 USA; ^5^ Department of Chemical Engineering Pohang University of Science and Technology (POSTECH) 77 Cheongam‐Ro, Nam‐Gu Pohang Gyeongbuk 37673 Republic of Korea; ^6^ Research Center O2MEDi inc. Ulsan 44919 Republic of Korea

**Keywords:** autophagy, drug‐resistance, Ir(III) complexes, oxidation, photodynamic therapy, protein modifications

## Abstract

Autophagy is a crucial quality control mechanism that degrades damaged cellular components through lysosomal fusion with autophagosomes. However, elevated autophagy levels can promote drug resistance in cancer cells, enhancing their survival. Downregulation of autophagy through oxidative stress is a clinically promising strategy to counteract drug resistance, yet precise control of oxidative stress in autophagic proteins remains challenging. Here, a molecular design strategy of biocompatible neutral Ir(III) photosensitizers is demonstrated, B2 and B4, for precise reactive oxygen species (ROS) control at lysosomes to inhibit autophagy. The underlying molecular mechanisms for the biocompatibility and lysosome selectivity of Ir(III) complexes are explored by comparing B2 with the cationic or the non‐lysosome‐targeting analogs. Also, the biological mechanisms for autophagy inhibition via lysosomal oxidation are explored. Proteome analyses reveal significant oxidation of proteins essential for autophagy, including lysosomal and fusion‐mediator proteins. These findings are verified in vitro, using mass spectrometry, live cell imaging, and a model SNARE complex. The anti‐tumor efficacy of the precise lysosomal oxidation strategy is further validated in vivo with B4, engineered for red light absorbance. This study is expected to inspire the therapeutic use of spatiotemporal ROS control for sophisticated modulation of autophagy.

## Graphical abstract

Autophagy inhibition via oxidative stress can overcome drug resistance in cancer, but the dynamic nature of autophagy makes this challenging. Biocompatible Ir(III) photosensitizers are designed to precisely control ROS at lysosomes and inhibit autophagy. Plausible mechanisms are established by proteomic and in vitro evidence. The therapeutic efficacy of the strategy is demonstrated in vivo with the red light‐absorbing photosensitizer, B4.

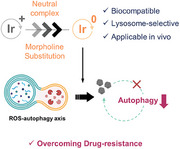

## 1 Introduction

Autophagy is a cellular process that mediates communication between lysosomes and autophagosomes for degradation and recycling of cellular components. It has emerged as a potent target for anti‐cancer therapy because autophagy can facilitate tumor survival by evading immune recognition,^[^ ^1^ ^]^ providing an energy source in nutrient‐poor conditions,^[^ ^2^ ^]^ and triggering drug resistance through supporting oncogenes and drug efflux.^[^ ^3^, ^4^, ^5^ ^]^ Therefore, several strategies, mostly small molecule inhibitors, have been developed to inhibit autophagy, sensitizing resistant cancer to chemotherapy.^[^ ^6^ ^]^ However, their efficacy can be easily hindered by genetic mutations at the binding site^.[^ ^7^ ^]^

**Figure 1:**
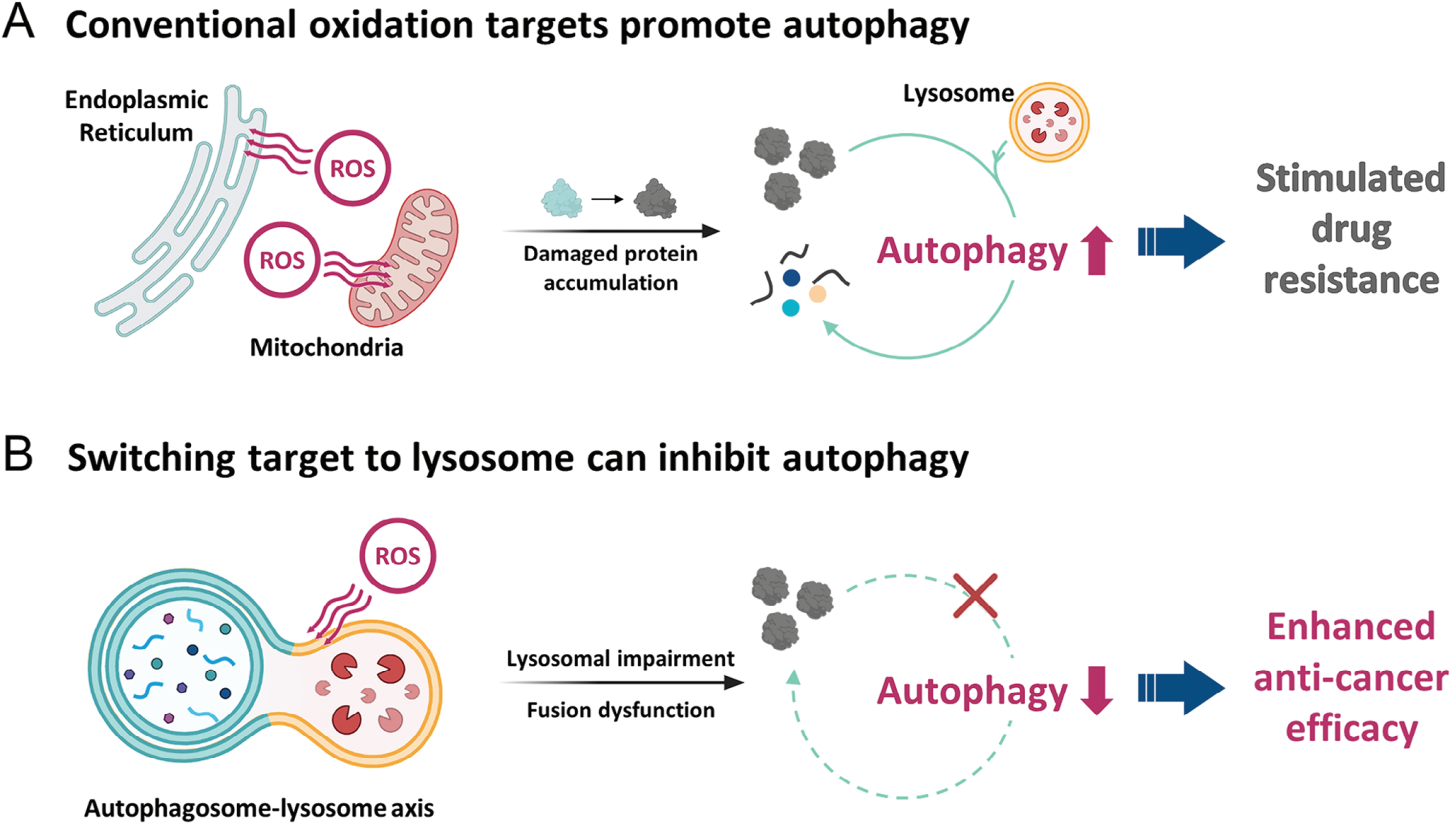
Schematic illustration of the dual role of reactive oxygen species (ROS) in autophagy. Oxidative stress can modulate autophagy in either a stimulatory or inhibitory manner. A) Oxidative stress at typical anti‐cancer targets, such as the endoplasmic reticulum and mitochondria, often promotes autophagy that can assist in tumor cell survival. B) In this work, we show that targeting acidic lysosomes for oxidative stress can effectively inhibit autophagy and enhance the anti‐cancer effect against drug‐resistant cancers. Lysosomal disruption and impaired autophagosome‐lysosome fusion prohibit the autophagic cycle.

One potent approach to overcome the secondary resistant mutations against protein binding inhibitors is to exploit reactive oxygen species (ROS).^[^ ^8^ ^]^ ROS are highly reactive molecules that can affect various cellular processes. Indeed, a recent clinical study showed that accumulated ROS could suppress autophagy and amplify the responsiveness of pancreatic ductal adenocarcinoma to chemotherapy.^[^ ^9^ ^]^ Nevertheless, exploiting ROS for autophagy inhibition remains a challenge, because ROS can act as either autophagy inducers or inhibitors depending on their amount and site of action.^[^ ^10^ ^]^ Oxidative stress at the endoplasmic reticulum (ER) and mitochondria often results in an elevation of autophagic flux due to accumulation of damaged proteins, as part of a cellular stress response (**Figure** **1**A).^[^ ^11^, ^12^, ^13^ ^]^ Thus, precise control of oxidative stress at the central organelles of autophagy is required for its therapeutic inhibition.^[^ ^14^ ^]^ However, the transient and dynamic nature of autophagy makes targeting specific autophagic proteins challenging. As an alternative tactic, pH‐sensitive protonation has been extensively proposed for targeting lysosomes with organic molecules,^[^ ^15^, ^16^, ^17^ ^]^ but these suffer from relative difficulty in synthesis, chemical instability, and low quantum yield (i.e., low oxidizing capacity). Although organometallic complexes have been developed to overcome these issues,^[^ ^18^ ^]^ their hydrophobic nature can lead to nonspecific binding with several proteins, potentially causing cytotoxicity.^[^ ^19^ ^]^ While most organometallic complexes have a cationic charge for enhanced water solubility and quantum yield,^[^ ^20^, ^21^ ^]^ this may hinder intended pH‐sensitive localization and biocompatibility.^[^ ^22^ ^]^ In contrast, neutral organometallic complexes face challenges in cellular uptake, photoluminescence quantum yield (PLQY, Φ_PL_), and targeting specific organelles.

Herein, we demonstrate a biocompatible molecular design strategy for spatiotemporal lysosomal oxidation. Our developed photosensitizers can utilize both the ROS‐autophagy axis and multimodal activity of PDT for overcoming drug‐resistant cancer in vivo (Figure 1B). To investigate lysosome selectivity and biocompatibility, we develop Ir(III) complexes, B1, B2, and C2. The morpholine‐substituted neutral Ir(III) complex, B2, is compared with the morpholine‐free analog, B1, to investigate lysosome selectivity, and the cationic analog, C2, to explore biocompatibility. B2 effectively targets lysosomes with minimal dark toxicity and induces cell death through photo‐controllable disruption of autophagy. Following proteomic analyses of the oxidized proteins suggest lysosome membrane permeabilization (LMP) and fusion malfunction, which are both strongly related to the prevention of autophagy. We confirm LMP using live cell microscopy, and fusion malfunction by SNARE model proteins. Furthermore, for in vivo photodynamic therapy (PDT), we modify B2 into B4 for red light absorption. The lysosomal oxidation by B4 shows anti‐cancer efficacy in mice bearing drug‐resistant pancreatic cancer (Panc‐1).

## 2 Results and Discussion

### 2.1 Lysosome‐Targeting Neutral Ir(III) Complex B2 Induces Oxidative Cell Death by Inhibiting Autophagy

**Figure 2:**
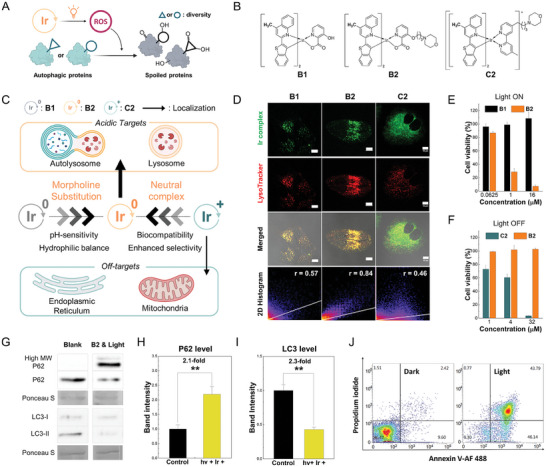
Lysosome‐specific and biocompatible B2 triggers photoinduced autophagy inhibition. A) Schematic illustration of photo‐induced autophagy inhibition via spatiotemporal reactive oxygen species (ROS) generation. ROS can destabilize proteins with a variety of structures or mutations. B) Chemical structures of B1, B2, and C2. C) Summary of molecular mechanism for lysosome‐specific biocompatible neutral Ir(III) complex with morpholine substitution. D) Localization patterns of B1, B2, and C2 analyzed using confocal microscopy and 2D histograms (green: Ir complex; red: LysoTracker probe; 2D Histogram: Pearson's coefficient). Lysosomes were stained with LysoTracker Red (50 nm) or LysoTracker Deep Red (50 nm) for 30 min after 2 h of incubation with 2 µm of Ir(III) complexes (Scale bar = 10 µm). E,F) Cytotoxicity of the Ir(III) complexes assessed using the 3‐(4,5‐dimethylthiazol‐2‐yl)‐2,5‐diphenyl tetrazolium bromide (MTT) assay. Phototoxicity and biocompatibility of B2 were compared with B1 and C2, respectively. (*n* = 4). G–I) Photoinduced inhibition of autophagy by B2 evaluated based on the expression level of P62 and LC3. The Ponceau S membrane staining is shown as a loading control. HeLa cells were treated with 8 µm B2 in DMSO with 90 s photoirradiation after 2 h of incubation. (*n* = 3, representative images shown). ^**^ *p* < 0.01. Data are presented as mean ± S.E.M. J) Apoptosis after B2 photosensitization was confirmed using flow cytometry.

We designed a photosensitizer, B2 that could induce focused damage on autophagic proteins at lysosomes and impair autophagy in a spatiotemporal manner (**Figure** **2**A,B). The lysosome‐targeting neutral iridium complex B2 was designed and successfully synthesized based on the following four criteria: light absorption in the visible light region, efficient ROS generation, high cellular toxicity under light irradiation, and low cytotoxicity under dark conditions (Figures S1–S10, Supporting Information). To enhance π‐conjugation length, we used (benzo[b]thiophen‐2‐yl) quinolone as the main ligand to accomplish visible light absorptivity.^[^ ^23^ ^]^ The functionalization of morpholine (lipophilic amine, pKa = 8.36) on the picolinate unit was considered because morpholine can be dominantly protonated and trapped in acidic lysosomes of pH lower than 4.5,^[^ ^24^ ^]^ and picolinate results in a neutral charge.

To investigate the structure‐activity relationship underlying effective lysosome localization, B2 was compared with its morpholine‐free analog and cationic analog, referred to as B1 and C2, respectively (Figure 2B). We assumed that the neutral charge of the Ir(III) complex would be important for the successful incorporation of the pH‐sensitive protonation moiety to organometallic complexes. This is because lipophilic cationic metal complexes commonly accumulate in mitochondria or endoplasmic reticulum, and can be easily trapped inside proteins or a cellular envelope.^[^ ^25^, ^26^, ^27^ ^]^ Such characteristics may hinder selective localization and stimulate unexpected cytotoxicity,^[^ ^22^ ^]^ which must be avoided for precise anti‐cancer therapy (Figure 2C). B1 and C2 were not expected to achieve biocompatible lysosomal localization, since B1 lacks an organelle‐directing group and C2 possesses 2,2′‐bipyridine, a typical ancillary ligand for cationic Ir(III) complexes.

The subcellular localization of B2 was analyzed in HeLa cells using confocal microscopy, and compared with B1 and C2, respectively (Figure 2D). B1 showed dot‐like patterns, possibly due to its lipophilicity, but was only partially localized in lysosomes with low selectivity. However, the morpholine‐substituted complex, B2, overlapped well with the LysoTracker probe signal, and showed higher Pearson's coefficient (r) than B1 (r_B1_ = 0.57 and r_B2_ = 0.84). Despite featuring morpholine and sharing the same main ligand, the cationic analog, C2, exhibited non‐specific localization patterns (r_C2_ = 0.46). The results suggest that adopting a neutral complex design is required to effectively utilize morpholine for enhanced selectivity toward lysosomes.

The phototoxicity and biocompatibility of B2 and its analogs were then evaluated in HeLa cells using the 3‐(4,5‐dimethylthiazol‐2‐yl)‐2,5‐diphenyl tetrazolium bromide (MTT) assay. After 1 min of white light exposure (0.6 J cm^−2^), B2 efficiently killed cancer cells at notably low concentrations, with an IC_50_^light^ value of 0.636 µm (Figure 2E). However, no photodynamic effect was observed for B1 at concentrations as high as 32 µm (Figure S18A, Supporting Information). This indicates that morpholine played crucial roles in therapeutic efficacy including precise localization and enhanced hydrophilic balance of the neutral compound.^[^ ^28^ ^]^ Furthermore, the undesired dark toxicity was significantly suppressed for B2 (Figure 2F; Figure S19, Supporting Information, IC_50_^dark, B2^ = 102 µm). In contrast, the cationic complex C2 showed increased dark toxicity derived from cationic character at relatively low concentrations (Figure S18B, Supporting Information, IC_50_^dark, C2^ = 9.24 µm), implying that neutral complex design was important for biocompatibility. Overall, B2 showed considerable potency for PDT with the phototoxicity index (PI, IC_50_^dark^/IC_50_^light^) of 161, which is 12 times higher than that of its cationic analog, C2 (PI_C2_ = 13.4). Also, the cell uptake amount of B2 was similar to B1 and threefold less than C2 (Figure S20, Supporting Information). These results suggest that the lysosome‐targeting neutral Ir(III) complex is a competent and biocompatible PDT agent.

Next, we measured the PLQY and the highest occupied molecular orbital and lowest unoccupied molecular orbital (HOMO/LUMO) energy levels of B2 using cyclic voltammetry and UV–vis spectroscopy. The Φ_PL_ of B2 was 0.49 and its HOMO/LUMO energy levels were −5.08/−2.94 eV, which are suitable for enhancing both electron and energy transfer to oxygen^[^ ^26^ ^]^ (Table S1 and Figures S21–S25, Supporting Information). To assess singlet oxygen (^1^O_2_, type II ROS) generation efficiency, 9,10‐anthracenediyl‐bis(methylene)dimalonic acid (ABDA) was used, while the dihydrorhodamine 123 assay was used to test radicals (O_2_^•−^and •OH, type I ROS). B2 efficiently produced both singlet oxygen and oxygen radicals (Figure S26, Supporting Information). The superior ROS production capability of B2 supported our design strategy for both electron and energy transfer. The ability to generate ROS was maintained at pH 4, with a slight increase in type I and a decrease in type II ROS (Figure S27, Supporting Information). This result suggests a propensity for lysosome‐specific oxidation. It is worth mentioning that B1 produces lower levels of ROS compared to B2, possibly due to different hydrophilic profiles provided by morpholine.^[^ ^28^, ^29^ ^]^

Encouraged by the specific lysosome localization and effective photodynamic effect of B2, we investigated the autophagy receptor p62 (SQSTM1) and autophagosome marker LC3‐I/II to determine whether the lysosome‐localized oxidative stress could hinder autophagy (Figure 2G; Refer to Figures S28 and S29, Supporting Information for the whole blot). An increase in p62 and a reduction in LC3 expression levels indicate the downregulation of autophagy via elevated oxidative stress.^[^ ^9^ ^]^ The total p62 abundance including a high molecular weight p62 band at >180 kDa increased 2.1‐fold after B2 photosensitization, although the abundance of p62 monomer at 55 kDa moderately decreased, possibly due to oxidation (Figure 2H). The high molecular weight band corresponds to polymerized p62, which also implies disturbed autophagy.^[^ ^30^ ^]^ This polymerization may have been induced by photo‐crosslinking due to the photocatalytic property of the Ir(III) photosensitizer.^[^ ^26^ ^]^ An increase in the total p62 band intensity suggests insufficient proteolysis of the recruited p62, indicating the prevalence of lysosome dysfunction. Along with p62 accumulation, LC3‐I/II levels were downregulated by 2.3‐fold, suggesting reduced autophagy as well (Figure 2I). In summary, lysosomal oxidative stress induced by B2 disturbed autophagic metabolism, as evidenced by the upregulation and crosslinking of p62 accompanied by LC3‐I/II downregulation. Ultimately, the inhibition of autophagy resulted in apoptotic cell death as analyzed by flow cytometry using Annexin V‐Alexa Fluor 488 and propidium iodide (Figure 2J). The following inhibitor‐based study further revealed complicated biological pathways involved in the cell death (Figure S30, Supporting Information). The pretreatment of a ferroptosis inhibitor liproxstatin‐1 failed to rescue cells from the oxidative cell death by B2. The result excludes ferroptosis, a representative caspase‐independent phenotype. A pan‐caspase inhibitor Z‐VAD‐FMK slightly improved cell viability but with no dramatic effect. This suggests that the inhibition of autophagy may promote a caspase‐independent signal once the caspase‐dependent pathways such as apoptosis or pyroptosis are blocked. Indeed, it was reported that enhanced autophagy protects cells from caspase‐independent cell death.^[^ ^31^ ^]^ However, cotreatment with the HSP90 inhibitor PU‐H71 restored cell viability to 60%. Given that HSP90 inhibition has been reported to stimulate chaperone‐mediated autophagy (CMA),^[^ ^32^ ^]^ the restored viability implies a potential involvement of CMA, in addition to macroautophagy, which was indicated by the expression levels of p62 and LC3.

### 2.2 Proteome‐Wide Analysis Reveals Severe Oxidative Damage to the Maintenance and Fusion of Lysosomes

Cellular damage follows diverse modes of action (MoA) depending on the location of ROS generation.^[^ ^33^ ^]^ In the mitochondria and ER, proteome‐wide analysis has determined the MoA underlying ROS‐induced cell death. However, no clear underlying mechanism for lysosome oxidation‐induced autophagy impairment has been provided until now. Therefore, we performed liquid chromatography‐tandem mass spectrometry (LC‐MS/MS) analysis to elucidate the proteome modifications induced by lysosomal oxidative stress. Proteins in which methionine (Met) was transformed into methionine sulfoxide (Met‐O) were analyzed using label‐free quantification (LFQ) based on peptide precursor intensity with quadruplicate data sets. Each experimental set consists of the following four groups depending on the presence of B2 and light (Ir±/hv±). Protein oxidation is expressed as a fold change (FC), which is the ratio of precursor intensity from the experimental group (Ir+/hv+) relative to the average intensity of the control groups (Ir−/hv−, Ir+/hv−, and Ir−/hv+ conditions). A significantly oxidized protein was defined as one in which at least two‐fold higher oxidation occurred in the Ir+/hv+ group than in the control groups (log_2_FC > 1) and the P‐value for reproducibility was less than 0.05 (−logP > 1.3).

**Figure 3:**
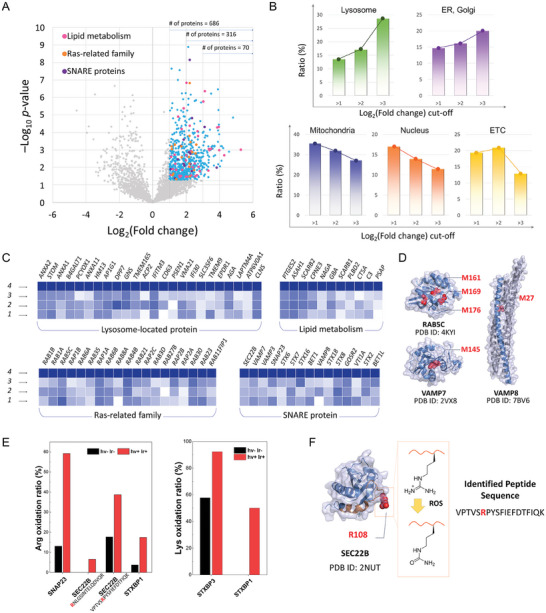
Oxidized proteome clues of oxidative damage in lysosomal maintenance and fusion. A–D) Oxidation of methionine residues: (A) Volcano plot of the oxidized proteome. The detected proteins with −logP > 1.3 and log_2_FC > 1 are shown in blue with the extra categories of lipid metabolism (magenta), Ras‐related family (orange), and SNARE proteins (purple). Stricter fold change cut‐off was also applied at log_2_FC = 2 and 3. (B) Percentage ratio of subcellular localization of the detected oxidized proteins at different cut‐off variations. (C) Heat maps depicting oxidation intensities of the significantly oxidized proteins. (lane 1: hv‐/Ir‐; lane 2: hv‐/Ir+; lane 3: hv+/Ir‐; lane 4: hv+/Ir+). (D) Crystal structures of the selected proteins and their Met‐oxidation sites. E) Ratio of oxidized amino acid sequences over the total identified sequences for arginine and lysine. F) Crystal structure of SEC22B with its oxidation site.

We identified 686 significantly oxidized proteins from the oxidized proteome profile (**Figure** **3**A). To validate significant oxidation at lysosomes, we analyzed the proportion of the oxidized lysosomal proteins depending on increments of the log_2_FC cut‐off value. Significant oxidation was observed in various organelles at log_2_FC > 1 because of the innate dynamic mobility of lysosomes,^[^ ^34^ ^]^ the relatively small proportion of lysosomal proteins,^[^ ^35^ ^]^ and the diffusion of ROS.^[^ ^36^ ^]^ However, at stricter log_2_FC cut‐off values, the oxidized protein ratio in lysosomes substantially increased from 14% (log_2_FC > 1) to 30% (log_2_FC > 3) (Figure 3B). This result indicates that oxidation mainly occurred proximal to lysosomes, which corresponds to the lysosome localization of B2 (Figure 2D). The oxidation ratios of the endoplasmic reticulum and Golgi apparatus were moderately increased at a high log_2_FC cut‐off value. This trend may be related to the close interactions of lysosomes with the endoplasmic reticulum and Golgi apparatus.^[^ ^37^ ^]^ To further assess lysosome‐focused oxidation and its distinctive MoA, we compared the heat map distribution of B2 with our previously reported proteome data of mitochondrial oxidation, which used an Ir(III) complex (Figure S31, Supporting Information).^[^ ^25^ ^]^ The apparent difference in heat maps indicated that the MoA of lysosomal oxidation differed from that of mitochondrial oxidation.

Next, we categorized the significantly oxidized proteins according to their functional clusters to elucidate the MoA of lysosomal oxidation‐induced cell death (Figure 3C). The two main categories were i) proteins involved in lipid metabolism and lysosome homeostasis and ii) proteins responsible for vesicle trafficking, such as Rab GTPases (Ras‐related family) and SNARE proteins. The Met‐O identified in lysosomal proteins and the proteins regarding lipid metabolism implicate damages to lysosomal membrane integrity. For example, deficiencies in lipogenetic proteins such as ASAH1, GBA, SCARB1, and PSAP may provoke LMP.^[^ ^38^ ^]^ Also, lysosomal membrane proteins are closely involved in the restoration of damaged lysosomes.^[^ ^39^ ^]^ This is noteworthy particularly for our system, as LMP is commonly associated with ROS.^[^ ^40^ ^]^ At the same time, the prevalent Met‐O observed in Rab GTPases and SNARE proteins, the key regulators of vesicle trafficking and fusion,^[^ ^41^, ^42^ ^]^ implies a malfunction in lysosomal fusion. For instance, the STX17‐SNAP29‐VAMP7 SNARE complex is required for autophagosome‐lysosome fusion.^[^ ^43^ ^]^

The crystal structures of RAB5c, VAMP7, and VAMP8 show the Met‐oxidation sites (red sphere) at α‐helix or β‐strand (Figure 3D). The oxidative dysfunction of SNARE and SNARE‐binding proteins was further supported by extra modifications of the arginine (Arg, R) and lysine residues (Lys, K) (Figure 3E; Figure S32, Supporting Information). Arg and Lys are important for the assembly of the “QQQR” core SNARE complex and for tight binding via ionic interactions.^[^ ^44^ ^]^ Thus, as exemplified by citrullination of Arg, a change in charge due to oxidation of Arg or Lys suggests hindered SNARE complex assembly (Figure 3F). In summary, the proteomic analysis suggests a collapse of lysosomal membrane integrity and malfunction of lysosomal fusion as the major paths of the autophagy inhibitory cell death by B2.

### 2.3 Lysosomal Membrane Permeabilization and Fusion Malfunction Shown In Vitro

**Figure 4:**
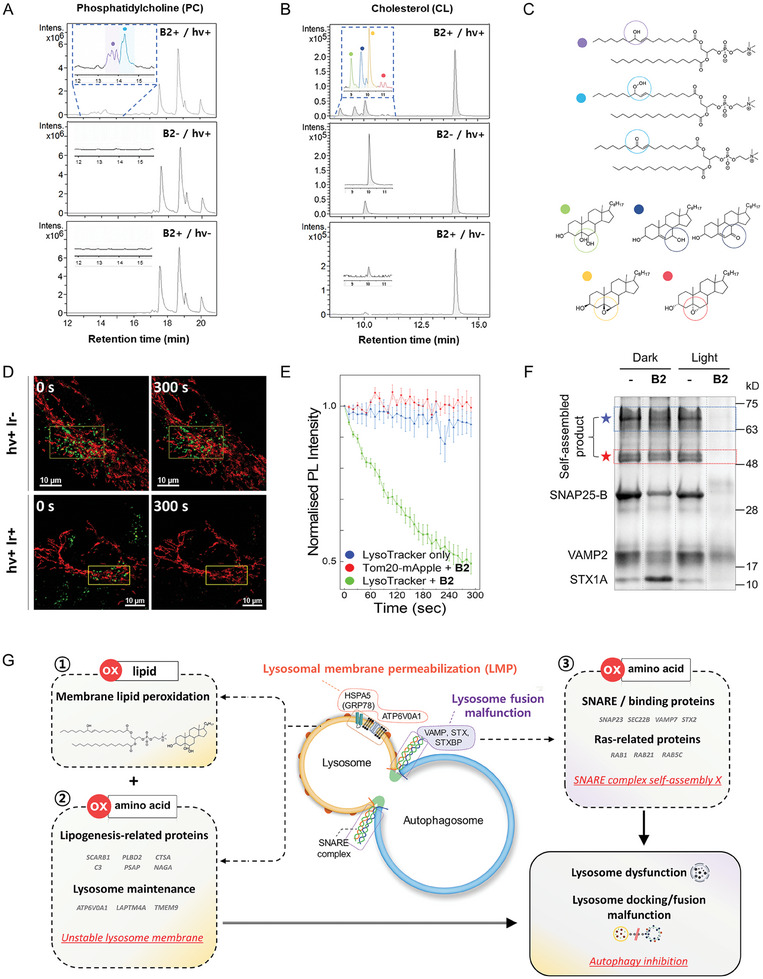
Lysosomal membrane permeabilization and SNARE assembly failure demonstrated in vitro. Identified products of A) phosphatidylcholine and B) cholesterol with or without in vitro B2 sensitization using LC‐MS/MS. C) Chemical structures of the assigned oxidative products. D) Confocal images of cells expressing Tom20‐mApple (red) and stained with LysoTracker (green). E) Chronological change in LysoTracker photoluminescence (PL) intensity with or without B2. Photobleaching was ruled out by simultaneous mApple tracking. Data are presented as mean values ± s.e.m. (*n* = 5). F) Coomassie Blue‐stained SNARE proteins and their complex in SDS‐PAGE. The self‐assembled SNARE complex (51.2 kDa) is denoted by the red star with oligomers (blue star). Each SNARE protein was treated with 500 µm B2 in DMSO and then photosensitized for 30 min. The proteins were then mixed and incubated at room temperature for 1 h to allow for SNARE complex formation. G) Plausible mode of action for oxidative inhibition of autophagy based on proteomic and in vitro evidence.

To verify the loss of lysosomal membrane integrity, lipid peroxidation, a typical cause of membrane permeabilization and disruption, was first examined in vitro.^[^ ^45^ ^]^ Two major lysosomal lipid species, phosphatidylcholine (PC) and cholesterol (Ch),^[^ ^46^ ^]^ were selected, and their oxidation by B2 photoactivation was assessed. The oxidized products of PC and Ch were characterized using LC‐MS/MS (**Figure** **4**A–C; Figures S33 and S34, Supporting Information). For both lipids, distinctive peaks were present after B2 photoactivation, which were identified as oxidized species of PC and Ch. The presence of oxidized lipid species suggests that photoexcited B2 localized in lysosomes can destabilize the enclosing membrane concurrently with protein oxidation.

As a result of the oxidation of lipids and lipogenetic proteins, fast lysosomal membrane disruption was monitored by the loss of lysosome probe signals (LysoTracker DeepRed; LTDR) in live cells. To verify specific damage to lysosomes, we used a cell line expressing fluorescent protein mApple at the mitochondrial membrane protein Tom20 as the control. The Tom20‐mApple intensity hardly changed with or without B2. However, a significant reduction of LTDR intensity was observed with B2 photoactivation (λ_ex_ = 514 nm) by laser, whereas no significant change in the LTDR signal was observed without B2 (Figure 4D,E). The loss of fluorescence intensity inside lysosomes with time was strongly indicative of the dynamic changes in the lysosomal environment, such as inconsistent pH or leakage of the probe, which were commonly associated with LMP.^[^ ^47^ ^]^ Furthermore, a sharp decline in lysosomal motility was observed while recording lysosome displacement, which implied a disruption of lysosome‐dependent metabolism (Figure S35, Supporting Information).^[^ ^48^ ^]^

We then explored the possible failure of lysosomal fusion caused by oxidation at the protein complex level. A highly stable self‐assembly of SNARE proteins embedded in membranes is the key driving force for overcoming the energetic barrier due to membrane–membrane repulsion barrier. It controls various cellular events, including lysosomal fusion to late endosome or autophagosome.^[^ ^49^ ^]^ We constructed a model SNARE complex that consists of three human SNARE proteins: syntaxin 1A (STX1A), synaptobrevin‐2 (VAMP2), and SNAP‐25B. The oxidative hindrance of the complex assembly and the decomposition of each protein were examined using SDS‐PAGE (Figure 4F; Figure S36, Supporting Information). Under no‐oxidation conditions (i.e., no B2 or light; lane 1, 2, and 3), the three SNARE proteins and their self‐assembled complexes (red or blue starred) were identified. In contrast, the abundance of the respective proteins and their complex were decreased after B2 photosensitization (the oxidation condition, lane 4): 3.72‐fold lower for the SNARE complex and 4.8‐, 1.75‐, and 1.54‐fold lower for STX1A, VAMP2, and SNAP‐25B, respectively (Figure S37, Supporting Information). The SNARE complex is highly stable and resistant to the harsh anionic detergent SDS.^[^ ^50^ ^]^ Thus, the substantial decrease in band intensities indicates the oxidative destabilization of the SNARE complexes. From the gel data, we inferred that the SNARE complex destabilization was likely caused by oxidative disruption of each SNARE proteins due to B2 photosensitization. This result is consistent with our mass spectrometry data that shows oxidative modifications on the amino acid residues critical to SNARE complex formation.

Based on our experimental data, we propose that autophagy can be perturbed via the spatiotemporal oxidation of proteins (Figure 4G). The process involves two routes. After the binding of morpholine to lysosomes by ion‐trapping, the first pathway directly impairs the lysosomal membrane through oxidation of lipids (①). The second pathway involves oxidative damage to proteins responsible for lipogenesis and membrane fusion/trafficking. The oxidation causes severe structural and functional impairment of the membrane maintenance and lipid metabolism‐related proteins (②). Damage to lipid metabolism‐related proteins such as ASAH1 and NAGA aggravates LMP, along with rapid membrane peroxidation. Protein oxidation also disturbs the processes required for successful membrane trafficking (③). Oxidized Rab GTPases perturb vesicle recruitment, and the impairment of SNAREs and their complex assembly hinders membrane docking and fusion. Significant oxidation of interactomes, such as those including Rab7 and VAMP7, hinders lysosomal fusion. As a result, lysosomal dysfunction at multiple levels disrupts autophagic metabolism, followed by apoptotic cell death.

### 2.4 Lysosomal Oxidation by Redshifted Ir(III) Complex Eradicates Drug‐Resistant Pancreatic Cancer In Vitro and In Vivo

**Figure 5:**
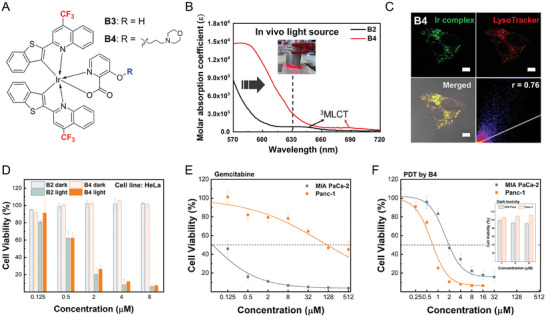
Efficacy of the redshifted Ir(III) complex B4 against drug‐resistant pancreatic cancer assessed in vitro. A) Chemical structures of B3 and B4. B) Bathochromic shift of absorbance of B4 compared with B2. Inset: 635 nm laser used for in vivo treatment. C) Subcellular localization of B4 (2 µm) colocalized with LysoTracker (50 nm). D) Photodynamic therapeutic potency of B4 comparable to B2 in HeLa cells. E) Sensitivity of MIA PaCa‐2 and Panc‐1 cell lines to gemcitabine. F) Sensitivity of MIA PaCa‐2 and Panc‐1 cell lines to B4 photosensitization. Inset: dark toxicity of B4 in the two cell lines. Data are presented as mean values ± s.e.m. (*n* = 4) for all MTT assay results.

To further evaluate the therapeutic efficacy of our strategy in vivo, we engineered B2 into a redshifted Ir(III) complex, B4. We introduced an electron withdrawing group ─CF_3_ into the main ligand instead of an electron donating ─CH_3_ (**Figure** **5**A; Figures S11–S17, Supporting Information). As a result, B4 displayed redshifted absorption compared to B2, with its triplet metal‐to‐ligand charge transfer (^3^MLCT) absorption tail reaching 690 nm that allows for improved tissue penetration (Figure 5B).^[^ ^51^ ^]^ The red‐absorbing Ir(III) complex exhibited comparable photostability to the commercially available PDT reagent, Photofrin, after 60 min of irradiation with a 630 nm LED, which corresponds to a high energy input of 180 J cm^−^^2^ (Figure S38, Supporting Information). B4 retained the ability to target lysosomes as confirmed by confocal microscopy owing to the substitution of morpholine (Figure 5C; Figure S39, Supporting Information). Electron paramagnetic spectroscopy confirmed its capability as both a type I and II ROS generator (Figure S40, Supporting Information). Importantly, B4 presented comparable PDT efficacy to B2 in HeLa cells, with IC_50_^light^ = 0.903 µm, IC_50_^dark^ = 106 µm, and PI = 118 (Figure 5D; Figures S41 and S42, Supporting Information). To test the potency of B4 against drug‐resistant cancer, MIA PaCa‐2 and Panc‐1 cells were chosen. Both cell lines are derived from pancreatic tumors but only Panc‐1 has been reported as an intrinsically chemo‐resistant cell line.^[^ ^52^ ^]^ Gemcitabine, a widely used chemotherapeutic agent for pancreatic cancer, demonstrated markedly less effectiveness in Panc‐1 cells (IC_50_ = 114.9 µm) compared to MIA PaCa‐2, where the IC_50_ was less than 0.125 µm (Figure 5E). On the other hand, B4 suppressed both cell lines less than threefold difference in IC_50_^light^ values (0.719 and 1.95 µm), maintaining biocompatibility under the dark at 16 µm (Figure 5F). This suggests that B4 can overcome intrinsic drug resistance in the cellular level.

**Figure 6:**
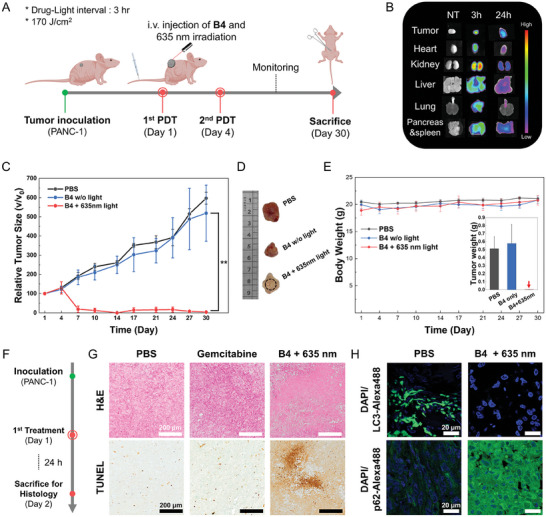
Efficacy of lysosome‐focused oxidation against drug‐resistant pancreatic cancer assessed in vivo. A) Schematic illustration of in vivo experiment. The photodynamic treatment with intravenously injected B4 and 635 nm laser (170 J cm^−2^) was conducted twice. B) Biodistribution study of BALB/c mice with Panc‐1 xenograft model 3 and 24 h post intravenous injection of B4 (10 mg kg^−1^). C) The change of tumor size (v/v_0_) for 30 days. Data are presented as mean values ± s.e.m. (*n* = 4), ^**^ *p* value < 0.01. D) The representative ex vivo tumor images on day 30 after sacrifice. E) The change of body weight. Inset: The weight of the tumor measured after sacrifice. F) Tumor tissue sample preparation schedule for histological analyses. G) Images of tumor tissues with H&E and TUNEL staining after the intravenous injection of treatment of PBS, gemcitabine (100 mg kg^−1^) or B4 (10 mg kg^−1^) (Scale bar = 200 µm) H) LC3 and p62 expression levels (green) in tumor tissues examined by immunohistochemical staining with or without B4 photosensitization (Blue: DAPI, Scale bar = 20 µm).

We further tested the anti‐cancer efficacy of B4 in a subcutaneous pancreatic cancer xenograft model in BALB/c mice bearing Panc‐1. Quadruple administration of 50 mg kg^−1^ gemcitabine decelerated Panc‐1 tumor growth by only 20% within the margin of error, necessitating a high dose of 100 mg kg^−1^ to achieve more than 50% tumor suppression (Figure S43, Supporting Information). Of note, a weekly dose of 50 mg kg^−1^ is generally considered efficacious, and the administration of 60 mg kg^−1^ gemcitabine twice was sufficient to inhibit tumor growth in MIA PaCa‐2 model by 59%.^[^ ^53^ ^]^ In contrast, PDT using B4 significantly reduced the tumor size with minimal undesirable toxicity. PDT with a 635 nm laser (170 J cm^−2^) was conducted twice on the 1st and 4th days, 3 h after each administration (**Figure** **6**A; Figure S44, Supporting Information). Biodistribution studies showed that intravenously injected B4 (10 mg kg^−1^) was efficiently delivered to the tumor within 3 h and accumulated there after 24 h (Figure 6B). The tumor size and body weight were monitored for 30 days following the first treatment. B4 effectively inhibited tumor growth only when irradiated with the 635 nm laser, implying its highly selective activity. The tumor size was significantly reduced after the 2nd PDT session, even eradicating the tumor in several cases (Figure 6C,D). Due to the biocompatibility and spatiotemporal reactivity of B4, it effectively reduced tumor weight with no significant body weight loss (Figure 6E). Moreover, minimal toxicity in liver and kidney was accomplished (Figure S45, Supporting Information). Histological analyses were conducted on Panc‐1 tumor samples collected 24 h after the first administration of 100 mg kg^−1^ gemcitabine or PDT with 10 mg kg^−1^ B4 (Figure 6F). A significant difference in necrosis and apoptosis of tumor tissue between gemcitabine and B4 treatments was shown by hematoxylin and eosin (H&E) stain and TUNEL assay, respectively (Figure 6G). Immunohistochemical analysis of LC3 and p62/SQSTM1 revealed downregulation of LC3 and upregulation of p62/SQSTM1 induced by B4, suggesting inhibition of autophagy in tumor tissue (Figure 6H). Overall, the lysosome‐focused oxidation by B4 demonstrated a promising tumor inhibition effect on chemotherapy‐resistant cancer with minimal toxicity.

## 3 Conclusion

We present a molecular design strategy to develop biocompatible PDT agents and highlight the effectiveness of chemically induced autophagy inhibition via lysosomal oxidation for overcoming drug‐resistant cancer. Our neutral Ir(III) photosensitizers, B2 and B4, selectively target lysosomes, and exhibit potent photodynamic therapeutic efficacy in 2D cancer cell cultures by downregulating autophagy with high biocompatibility. Proteomic and in vitro analyses confirm the possible relationship between spatiotemporal protein oxidation and autophagy inhibition. We find that lysosomal protein oxidation can impair maintenance of lysosomal membrane integrity and SNARE‐mediated membrane fusion, which are essential for autophagic processes. Furthermore, the red‐light activatable Ir(III) complex, B4, can be successfully applied to drug‐resistant cancer in vivo. Our study demonstrates that oxidative stress can be chemically controlled for therapeutic inhibition of autophagy by targeting lysosomes. The molecular design strategy and autophagy inhibition mechanism involving protein oxidation presented here are expected to offer new insights into the ROS‐autophagy axis as a potential target for anti‐cancer therapy. Future research should focus on discovering the specific proteins among the identified proteome that serve as key regulators toward the precise oxidative modulation of autophagy, and assess the extent to which autophagy‐derived resistance influences the therapeutic efficacy.

## 4 Experimental Section

### LC‐MS/MS

After staining with colloidal Coomassie blue, the protein gels were sliced in six consecutive portions followed by in‐gel tryptic digestion, as described in Supporting Information. The resulting tryptic peptides were analyzed by LC‐MS/MS. All mass analyses were performed on a Q Exactive Plus orbitrap mass spectrometer (Thermo Fisher Scientific, MA, USA) equipped with a nanoelectrospray ion source. To separate the peptide mixture, a C18 reverse‐phase HPLC column (500 mm × 75 µm ID) was used with an acetonitrile/0.1% formic acid gradient from 2.4 to 24% for 120 min at a flow rate of 300 nL min^−1^. For MS/MS analysis, the precursor ion scan MS spectra (m/z 400–2000) were acquired in the Orbitrap at a resolution of 70 000 at m/z 400 with an internal lock mass. The 20 most intensive ions were isolated and fragmented by high‐energy collision‐induced dissociation.

### LC‐MS/MS Data Processing

All MS/MS samples were analyzed using the Sequest Sorcerer platform (Sagen‐N Research, San Jose, CA, USA). Sequest was set up to search the Homo sapiens (20 612 entries, UniProt (http://www.uniprot.org)), which includes frequently observed contaminants, assuming the digestion enzyme trypsin. Sequest was searched with a fragment ion mass tolerance of 1.00 Da and parent ion tolerance of 10.0 ppm. Carbamidomethyl of cysteine was specified in Sequest as a fixed modification. Oxidation of methionine and acetyl of the N‐terminus were specified in Sequest as variable modifications. To search for another oxidation of Arg and Lys, R1 (N‐hydroxyl arginine), R2 (citrulline), R3 (glutamic semialdehyde), R4 (unknown structure), K1 (hydroxyl lysine), K2 (aminoadipic semialdehyde), and K3 (aminoadipic acid) were specified in Sequest as additional variable modifications. Scaffold Q+ (version 5.0.1, Proteome Software Inc., Portland, OR) was used to validate MS/MS‐based peptide and protein identifications. Peptide identifications were accepted if they could be established at a probability greater than 99.0% to achieve an FDR lower than 1.0% using the Scaffold Local FDR algorithm.^[^ ^54^ ^]^ Protein identifications were accepted if they could be established at a probability greater than 14.0% to achieve an FDR lower than 1.0% and contained at least two identified peptides. Protein probabilities were assigned by the Protein Prophet algorithm. Proteins that contained similar peptides and could not be differentiated based on MS/MS analysis alone were grouped to satisfy the principles of parsimony. Proteins were annotated with GO terms from NCBI (downloaded Feb 05, 2020).^[^ ^55^ ^]^ Based on proteome data, the total precursor intensity value was utilized for LFQ of amino acid oxidations. Normalization was performed iteratively (across samples) on intensities. Medians were used for averaging. Of 1 483 749 spectra in the experiment at the given thresholds, 565 863 (38%) were included in quantitation. Spectra data were log base2‐transformed, pruned of those matched to multiple proteins, and not reproducibly detected values were filled by imputed values representing a normal distribution around the detection limit. Based on the total matrix, a new distribution was created by Gaussian distribution with a downshift of 1.8 and width of 0.3 standard deviations. All processes were progressed using the Perseus software platform from the Max Planck Institute of Biochemistry.

## Conflict of Interest

The authors declare no conflict of interest.

## Supporting information



Supporting Information

Supporting Information

Supplemental Video 1

Supplemental Video 1

## Data Availability

The data that support the findings of this study are available in the supplementary material of this article.
