# Supplementary material for: Rational Design of Biocompatible Ir(III) Photosensitizer to Overcome Drug‐Resistant Cancer via Oxidative Autophagy Inhibition
Source: Adv Sci (Weinh). 2024 Nov 14;12(2):2407236. doi: 10.1002/advs.202407236 (PMC11727131; doi:10.1002/advs.202407236)
Supplement: Supplementary file 2 — Supporting Information [file ADVS-12-2407236-s001.docx]

Supporting Information

Rational Design of Biocompatible Ir(III) Photosensitizer to Overcome Drug-Resistant Cancer via Oxidative Autophagy Inhibition

Mingyu Park, Jung Seung Nam, Taehyun Kim, Gwangsu Yoon, Seoyoon Kim, Chaiheon Lee, Chae Gyu Lee, Sungjin Park, Kochan S. Bejoymohandas, Jihyeon Yang, Yoon Hee Kwon, Yoo Jin Lee, Jeong Kon Seo, Duyoung Min*, Taiho Park*, and Tae-Hyuk Kwon*

**Content**

**1. Experimental section**

**Materials and characterizations**

**Synthesis and spectral data**

**Cyclic voltammetry (CV)**

**Computational methods**

**Photophysical characterization**

**Singlet oxygen (^1^O_2_) detection in aqueous solution**

**Superoxide radical anion (O_2_^•-^)** **detection in aqueous solution**

**UPLC-HRMS analysis for lipid oxidation products**

**Cell culture and cell study**

**Subcellular localization and phenotype imaging**

**Cell viability assay (MTT assay)**

**Intracellular uptake of Ir complexes (ICP-OES)**

**Western blot analysis of autophagic flux related proteins (LC3-I/II, P62)**

**Quantification of western blot signal intensity**

**Flow cytometry analysis by fluorescence-activated single cell sorting**

**SNARE protein expression and purification**

**Preparation of tryptic peptides for LC-MS/MS**

**Electron paramagnetic resonance (EPR) spectroscopy**

**Tumor modeling and in vivo assessment of antitumor effect**

**Liver and kidney toxicity evaluation**

**Microscopic histochemistry**

**2. Supporting data**

**Figure S1–S45**

**Table S1 and S2**

**3. References**

**1. Experimental section**

**Materials and characterizations.** A 500 MHz Bruker NMR spectrometer was used to record the ^1^H, ^13^C and ^19^F NMR spectra of the iridium(III) complex in CDCl_3_ solution. The chemical shifts (*δ*) of the signals are given in ppm and referenced to the internal standard tetramethylsilane [Si(CH_3_)_4_]. The signals splitting is abbreviated as follows: *s* = singlet; *d* = doublet; *t* = triplet; *dd* = doublet of doublets; *dq* = doublet of quintets; *td* = triplet of doublets; *m* = multiplet. Coupling constants (*J*) are given in Hertz (Hz). 2-Chloro-4-methylquinoline, 4-trifluoromethyl-2(1H)-quinolinone, phosphoryl chloride, sodium hydroxide, benzo[b]thiophene-2-boronic acid, tetrakis(triphenylphosphine)palladium(0), sodium carbonate, IrCl_3_•x(H_2_O), 2-ethoxyethanol, 3-hydroxypicolinic acid, 4-(3-chloropropyl)morpholine, 4,4'-dimethyl-2,2'-bipyridine, 1,3-dibromopropane and morpholine were employed in the synthesis of the iridium(III) complex (**B1-4 and C2**). These chemicals were purchased from Sigma Aldrich and were used without any further purification. Firstly, the cyclometalated ligands, namely, 2-(benzo[b]thiophen-2-yl)-4-methylquinoline [btmq] (**L1**) and 2-(benzo[b]thiophen-2-yl)-4-(trifluoromethyl)quinoline [bttmq] (**L2**) were synthesized through the Suzuki-Miyaura cross coupling reaction and fully characterized by the method in our earlier reports.^2, 3^ Secondly, the synthesis of the iridium dimer complex [(C^∧^N)_2_Ir(μ-Cl)]_2_ was carried out by the reaction of IrCl_3_•x(H_2_O) and cyclometalating ligands (**L1** and **L2**) in a mixture of 2-ethoxyethanol and water, which is well known as the Nonoyama reaction.^2^ The synthesis of 4-(4-bromobutyl)-4'-methyl-2,2'-bipyridine was conducted according to literature.^3^ Thin-layer chromatography (TLC) was used to monitor the reaction progress (silica gel 60 F254, Merck Co.) and the spots were observed under UV light at 254 and 365 nm. Silica column chromatography was performed using silica gel (230–400 mesh, Merck Co.). The dry solvents are purified using J.C. Metyer solvent drying system. All other reagents are of analytical grade and used as received from Sigma Aldrich, Alfa Aesar, Tokyo Chemical Industry and Samchun chemicals unless otherwise specified.

**Synthesis and spectral data**

*General synthetic procedure for complexes B1 and B3***.** A mixture of one equivalent of the corresponding dimer complex [(C^∧^N)_2_Ir(μ-Cl)]_2_, 2.6 equivalents of 3-hydroxypicolinic acid and 11 equivalents of sodium carbonate were stirred overnight in a 3:1 mixture of dichloromethane and ethanol (40 mL) at 70 °C under argon atmosphere. The solvent was removed by evaporation under reduced pressure. The crude product obtained was poured into water and extracted with ethyl acetate (3 × 50 mL). The combined organic layer was dried over Na_2_SO_4_. The solvent was removed under reduced pressure to give a crude residue. The crude product was purified by using silica gel column chromatography with CH_2_Cl_2_:methanol in 9:1 ratio as eluent, giving the desired complex as dark red powder (for **B1**) and black powder (for **B3**) All purified samples were recrystallized and vacuum dried before conducting all analysis.

*Spectral data of bis[2-(benzo[b]thiophen-2-yl)-4-methylquinoline]iridium(III) (3-hydroxy picolinate): (btmq)_2_Ir(3-HPA) (B1).* Yield: 52%. B1, C_42_H_28_IrN_3_O_3_S_2_, ^1^H NMR (500 MHz, Chloroform-d) δ = 13.14 (s, 1H), 8.33 – 8.28 (m, 1H), 7.89 – 7.77 (m, 3H), 7.76 – 7.67 (m, 3H), 7.61 (dd, J = 5.1, 1.4 Hz, 1H), 7.43 – 7.33 (m, 2H), 7.26 – 7.07 (m, 4H), 6.98 (dtd, J = 7.0, 3.6, 1.3 Hz, 2H), 6.73 (ddd, J = 8.6, 6.9, 1.5 Hz, 1H), 6.69 – 6.63 (m, 2H), 6.50 (ddd, J = 8.1, 7.0, 1.1 Hz, 1H), 6.04 (d, J = 8.0 Hz, 1H), 2.92 (dd, J = 3.8, 0.9 Hz, 6H). ^13^C NMR (151 MHz, CDCl_3_) δ = 171.88, 166.95, 165.38, 152.93, 152.47, 150.69, 149.10, 148.08, 147.69, 147.09, 146.94, 146.09, 142.82, 142.63, 140.24, 137.88, 137.84, 131.71, 129.07, 127.49, 127.37, 127.23, 126.74, 126.64, 126.21, 125.85, 125.61, 125.20, 124.93, 124.75, 124.43, 124.10, 123.63, 123.44, 122.54, 122.48, 119.17, 117.89, 19.17, 19.06.

*Spectral data of bis[2-(benzo[b]thiophen-2-yl)-4-(trifluoromethyl)quinoline]iridium(III) (3-hydroxy picolinate): (bttmq)_2_Ir(3-HPA) (B3).* Yield: 53%. B3, C_42_H_22_F_6_IrN_3_O_3_S_2_, ^1^H NMR (500 MHz, Chloroform-d) δ = 12.96 (s, 1H), 8.36 – 8.30 (m, 1H), 8.12 (d, J = 6.4 Hz, 2H), 8.02 (d, J = 8.4 Hz, 1H), 8.00 – 7.96 (m, 1H), 7.85 (d, J = 8.1 Hz, 1H), 7.76 – 7.72 (m, 1H), 7.53 (dd, J = 5.2, 1.3 Hz, 1H), 7.51 – 7.44 (m, 2H), 7.33 (ddd, J = 8.2, 6.9, 1.1 Hz, 1H), 7.25 (d, J = 5.2 Hz, 1H), 7.23 – 7.17 (m, 2H), 7.11 – 7.07 (m, 1H), 7.06 (ddd, J = 8.1, 7.0, 1.2 Hz, 1H), 6.84 (ddd, J = 8.6, 6.8, 1.3 Hz, 1H), 6.74 (ddd, J = 8.1, 7.0, 1.1 Hz, 1H), 6.64 (dt, J = 8.1, 1.0 Hz, 1H), 6.56 (ddd, J = 8.2, 7.0, 1.1 Hz, 1H), 5.98 (dt, J = 8.2, 0.9 Hz, 1H). ^13^C NMR (151 MHz, Chloroform-d) δ = 176.10, 167.18, 165.60, 159.66, 154.94, 153.26, 150.06, 149.21, 146.39, 146.31, 143.27, 143.02, 140.44, 138.14, 137.37, 135.09, 132.84, 130.58, 129.37, 127.46, 127.41, 127.33, 126.47, 126.45, 125.77, 125.29, 124.76, 124.50, 124.27, 124.13, 123.68, 122.97, 122.90, 121.86, 120.98, 120.09, 116.20, 114.74. ^19^F NMR (471 MHz, Chloroform-d) δ = -60.46, -60.57.

*General synthetic procedure for complexes B2 and B4.* A mixture of one equivalent of the corresponding iridium complex (B1 and B3), 5 equivalents of 4-(3-chloropropyl)morpholine and 15 equivalents of sodium carbonate were stirred overnight in dry N,N-dimethylformamide at 140 °C under argon atmosphere. The solvent was removed by evaporation under reduced pressure. The crude product obtained was poured into water and extracted with ethyl acetate (3 × 50 mL). The combined organic layer was dried over Na_2_SO_4_. The solvent was removed under reduced pressure to give a crude residue. The crude product was purified by using silica gel column chromatography with CH_2_Cl_2_:methanol in 9:1 ratio as eluent, giving the desired complex as red powder (for **B2**) or dark red powder (for **B4**) All purified samples were recrystallized and vacuum dried before conducting all analysis.

*Spectral data of bis[2-(benzo[b]thiophen-2-yl)-4-methylquinoline]iridium(III) [3-(4-morpholinyl)propoxy]picolinate: (btmq)_2_Ir(mor-pic) (B2).* Yield: 54%. B2, C_49_H_41_IrN_4_O_4_S_2_, ^1^H NMR (500 MHz, Chloroform-d) δ = 8.56 (d, J = 8.6 Hz, 1H), 7.87 – 7.72 (m, 5H), 7.72 – 7.64 (m, 2H), 7.42 – 7.29 (m, 2H), 7.24 – 7.04 (m, 4H), 6.99 – 6.89 (m, 2H), 6.71 – 6.60 (m, 3H), 6.47 (t, J = 7.6 Hz, 1H), 5.98 (d, J = 8.2 Hz, 1H), 3.93 – 3.80 (m, 2H), 3.57 (t, J = 4.7 Hz, 4H), 2.90 (d, J = 11.7 Hz, 6H), 2.30 (s, 6H), 1.84 (h, J = 6.8 Hz, 2H). ^13^C NMR (151 MHz, Chloroform-d) δ = 176.41, 170.25, 166.96, 165.62, 157.98, 154.49, 150.86, 149.37, 148.24, 147.61, 147.54, 147.25, 146.93, 142.77, 142.72, 141.20, 140.01, 139.00, 138.01, 131.61, 129.41, 129.11, 127.86, 127.48, 126.92, 126.58, 126.43, 126.37, 126.21, 126.13, 125.83, 125.71, 125.62, 125.50, 125.16, 124.92, 124.55, 124.46, 124.41, 124.11, 124.00, 123.60, 123.52, 123.39, 122.58, 122.50, 122.44, 119.16, 119.05, 117.97, 68.34, 66.01, 55.02, 53.21, 30.86, 19.15, 19.03. (ESI-MS) [C_49_H_41_IrN_4_O_4_S_2_]: calcd, m/z = 1006.23; found, m/z = 1007.42 [M+].

*Spectral data of bis[2-(benzo[b]thiophen-2-yl)-4-(trifluoromethyl)quinoline]iridium(III) [3-(4-morpholinyl)propoxy]picolinate: (bttmq)_2_Ir(mor-pic) (B4).* Yield: 57%. B4, C_49_H_35_F_6_IrN_4_O_4_S_2_, ^1^H NMR (500 MHz, Chloroform-d) δ = 8.59 (d, J = 8.4 Hz, 1H), 8.13 (d, J = 15.0 Hz, 2H), 8.00 (dd, J = 22.2, 8.1 Hz, 2H), 7.87 (d, J = 8.1 Hz, 1H), 7.77 – 7.68 (m, 2H), 7.47 (tt, J = 7.1, 5.5 Hz, 2H), 7.32 (d, J = 3.8 Hz, 3H), 7.20 (t, J = 7.6 Hz, 1H), 7.06 (q, J = 8.3, 7.9 Hz, 2H), 6.81 (t, J = 7.8 Hz, 1H), 6.74 (t, J = 7.6 Hz, 1H), 6.64 (d, J = 8.0 Hz, 1H), 6.60 – 6.51 (m, 1H), 5.95 (d, J = 8.1 Hz, 1H), 3.95 (s, 2H), 3.61 (s, 4H), 2.38 (s, 6H), 1.92 (s, 2H). ^13^C NMR (151 MHz, CDCl_3_) δ = 169.89, 167.18, 165.84, 158.37, 158.12, 153.95, 150.45, 149.24, 146.72, 146.42, 143.27, 143.24, 140.40, 140.10, 138.62, 138.36, 137.08, 136.85, 136.63, 132.73, 130.39, 128.52, 127.49, 127.34, 127.21, 126.95, 126.34, 126.18, 125.74, 125.58, 125.23, 124.63, 124.44, 123.98, 123.76, 122.89, 122.82, 121.94, 120.93, 120.07, 116.27, 114.72, 68.11, 66.86, 66.62, 58.78, 53.66, 53.61, 53.10, 29.66, 26.92.^19^F NMR (471 MHz, Chloroform-d) δ = -60.36, -60.65. (ESI-MS) [C_49_H_35_F_6_IrN_4_O_4_S_2_]: calcd, m/z = 1114.17; found, m/z = 1115.25 [M+].

*Synthesis of* *4-(4-(4'-methyl-[2,2'-bipyridin]-4-yl)butyl)morpholine.* 4-(4-Bromobutyl)-4'-methyl-2,2'-bipyridine was prepared by literature method^8^. For the morpholine substitution, a mixture of 1 equivalent of 4-(4-Bromobutyl)-4'-methyl-2,2'-bipyridine, 1.5 equivalent of K_2_CO_3_, 5 equivalents of morpholine, and acetonitrile was refluxed under inert Ar condition at 100 ^o^C for 20 hours. The reaction crude was extracted with CH_2_Cl_2_ and brine, and purified by using silica gel column chromatography with ethyl acetate.

*Synthesis of bis[2-(benzo[b]thiophen-2-yl)-4-methylquinoline]iridium(III)[ 4-(4-(4'-methyl-[2,2'-bipyridin]-4-yl)butyl)morpholine]: (btmq)_2_Ir(mor-bpy) (C2).* A mixture of one equivalent of the corresponding dimer complex [(C^∧^N)_2_Ir(μ-Cl)]_2_ and 1.9 equivalents of 4-(4-(4'-methyl-[2,2'-bipyridin]-4-yl)butyl)morpholine were refluxed overnight in 1:1 solution of dry methanol and CH_2_Cl_2_ under argon atmosphere. The solvent was removed by evaporation under reduced pressure and redissolved in ethanol. The residual solid was filtered out, and the filtrate was evaporated in vacuo. The product was obtained by triturating with CH_2_Cl_2_ and hexane and washed with extra hexane. ^1^H NMR (400 MHz, DMSO) δ 8.26 (d, J = 23.8 Hz, 2H), 8.15 – 8.04 (m, 4H), 7.95 (dd, J = 18.3, 8.1 Hz, 4H), 7.56 (t, J = 6.2 Hz, 2H), 7.32 (t, J = 6.2 Hz, 2H), 7.12 (t, J = 7.3 Hz, 2H), 6.92 (dt, J = 25.6, 8.1 Hz, 4H), 6.63 (dd, J = 14.3, 7.0 Hz, 2H), 6.18 (dd, J = 22.8, 8.2 Hz, 2H), 3.52 – 3.46 (m, 4H), 2.89 (s, 3H), 2.88 (s, 3H), 2.64 (t, J = 7.3 Hz, 2H), 2.38 (s, 3H), 2.16 (dd, J = 19.2, 12.3 Hz, 6H), 1.55 – 1.46 (m, 2H), 1.22 – 1.14 (m, 3H). 13C NMR (101 MHz, cdcl3) δ 165.69, 157.02, 156.00, 155.66, 153.15, 153.02, 149.12, 149.10, 148.18, 148.06, 146.64, 146.21, 145.97, 142.79, 139.86, 139.82, 130.75, 130.52, 128.08, 127.22, 127.14, 126.43, 126.36, 126.29, 126.01, 125.97, 124.98, 124.90, 124.32, 124.26, 124.15, 122.79, 118.66, 77.32, 77.00, 76.69, 66.50, 58.40, 53.44, 34.57, 27.72, 25.41, 21.28, 19.19. (ESI-MS) [C_55_H_49_IrN_5_OS_2_]: calcd, m/z = 1052.30; found, m/z = 1052.2999 [M+].

**Cyclic voltammetry (CV).** Cyclic voltammetry experiments were carried out with a PowerLab/AD instrument model system using three electrode cell assemblies. Platinum wires were used for counter electrodes, a silver wire was used as Ag/Ag^+^ quasi reference electrode and a platinum electrode was used as a working electrode. Measurements were carried out in dichloromethane solution with tetrabutylammonium hexafluorophosphate as supporting electrolyte at a scan rate of 100 mV/s. Concentrations of iridium(III) complex and supporting electrolyte were 5×10^-3^ and 0.2 M, respectively. The ferrocenium/ferrocene couple (FeCp_2_^+^/FeCp_2_^0^) was used as an internal reference. The energy level of FeCp_2_^+^/FeCp_2_^0^ was assumed at –4.8 eV to vacuum.^3^ All solutions for the electrochemical studies were deaerated with pre-purified argon gas prior to the measurements.

**Computational methods.** The geometrical structures, transition dipole moments and frontier molecular orbitals (FMOs) of the highest occupied molecular orbital (HOMO) and lowest occupied molecular orbital (LUMO) were optimized by using density functional theory (DFT) based on a method with Perdew-Burke-Ernzerhof (PBE0) hybrid functional with LANL2DZ basis set for the iridium (Ir) atom and 6-31G* for the rest of the atoms. All calculations were carried out with Gaussian 09 package.

**Photophysical characterization.** The electronic absorption spectrum of the iridium(III) based complexes was measured on a Mecasys Optizen Pop UV/vis spectrophotometer. For the photoluminescence (PL) spectroscopy analysis, solutions were recorded on a spectrofluorometer/phosphorimeter (FluoroMax Plus-C-P, HORIBA) with a solid state laser in continuous wave operation at an excitation power of 150W ozone free xenon lamp sources. The absolute luminescence quantum efficiencies in the solution states were measured using 3.2-inch internal integrating sphere (FM-Sphere, HORIBA). Phosphorescence lifetimes were measured using time correlated single photon counting (TCSPC) system (HAMAMATSU/C11367-31).


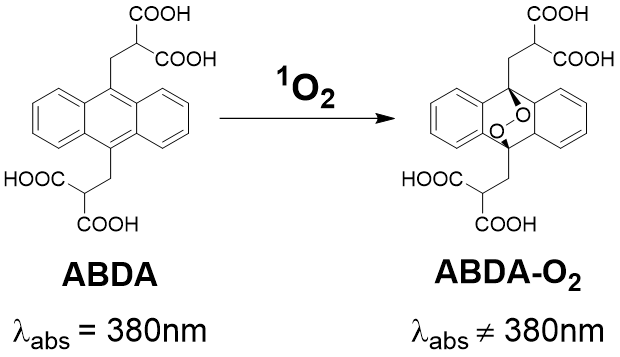
**Singlet oxygen (^1^O_2_) detection in aqueous solution.** Singlet oxygen (^1^O_2_) generation was measured by determining absorbance decay of ^1^O_2_ indicator 9,10-anthracenediyl-bi(methylene)dimalonic acid (ABDA). In the presence of ^1^O_2_, peroxide bridge is formed at the anthracene core of ABDA and the initial 380nm absorbance decays. Each 10μM **B2** and **B4** solutions was prepared in DMSO/H_2_O=1:99 solvent. 100mM stock solution of ABDA in DMSO was then added to as prepared iridium solution to 1:1000 volume ratio. The final solutions were irradiated with a LED (λ_emission_=520nm, 1.87mW/cm^2^) for 0 to 8 minutes. Then, ABDA absorbance decay was evaluated by measuring (Abs _sample_ – Abs _background_) at 380nm by microplate reader.

**Superoxide radical anion (O_2_^•-^)** **detection in aqueous solution.** Superoxide radical anion (O_2_^•-^) generation was evaluated by measuring fluorescence intensity of Rhodamine 123 which is the reaction product between O_2_^•-^ and Dihyrorhodamine123 (DHR123). The formation of rhodamine was monitored at 530nm using fluorometer. 10μM **B2** and **B4** solutions was prepared in DMSO/H_2_O=1:99 solvent and was added with 4mM stock solution of DHR123 in DMSO to 1000:1 volume ratio. The final solutions were irradiated with a LED (λ_emission_=520nm, 1.87mW/cm^2^) for 0 to 8 minutes. Then, fluorescence of rhodamine 123 was recorded by microplate reader. (λ_excitation_ = 507 nm, λ_emission_ = 530 nm)

**UPLC-HRMS analysis for lipid oxidation products.** The phosphatidylcholine (**PC**) and cholesterol (**Ch**) oxidation by photosensitization of **B2** were investigated by UPLC-HRMS analysis. The **PC** and **B2** were dissolved to EtOH-H_2_O solvent (EtOH 30% v/v) to prepare **PC** sample solutions ([**PC**] = 1 mM, [**B2**] = 100 μM), then the **PC** samples were irradiated with a photoreactor LED (λ = 525-530 nm, 10 mW/cm^2^ for 2 hours) (HepatoChem, HCK1012-01-004). After irradiation, the solvent was removed under reduced pressure. The **PC** samples were dissolved to MeOH and then injected into ultra-high performance liquid chromatography (UPLC) (ACQUITY I CLASS, WATERS/Q-TOF) system. A WATERS BEH C8 column (2.1 X 100, 1.7 μm) was employed, and samples were eluted with a water/acetonitrile. The %acetonitrile was kept at 63% for the initial 10 minutes, increasing to 100% at 22 minutes. Then, the %acetonitrile was kept until 27 min. At 28 min, %acetonitrile was back to 63%, and this percentage was kept until the end of the run at 30 min. A High-resolution mass spectrometry (HRMS) (maxis HD, Bruker) controlled by the software smartformula was employed for mass analysis. The mass spectra were detected by electrospray ionization (ESI) in the positive mode, with the following conditions: capillary voltage: 4500 V; charging voltage: 2000 V; dry gas temperature: 200 ^o^C; mass range: 50-3000 m/z. The m/z value of each peak of chromatogram were assigned as following: 760.5851 = [**PC**+H]^+^; 774.5643 = [**PC**-ketone+H]^+^; 776.5800 = [**PC**-alcohol+H]^+^; and 792.5749 = [**PC**-peroxide+H]^+^. The **Ch** oxidation analysis was carried out as well. The **Ch** and **B2** were dissolved to EtOH-H_2_O solvent (EtOH 70% v/v) to prepare **Ch** sample solutions ([**Ch**] = 1 mM, [**B2**] = 100 μM), then the solution was irradiated with the same condition above. After irradiation, the solvent was removed under reduced pressure. The **Ch** samples were dissolved to MeOH, then injected into the UPLC system. Using the WATERS BEH C8 column (2.1 X 100, 1.7 μm), the **Ch** samples were eluted with a water/MeOH. The %MeOH was held on 80% for the initial 0.5 min, rising to 100% at 15 min. The percentage was kept until 19 min, then back to 80% until 19.5 min. The %MeOH was kept until the end of the run at 22 min. HRMS spectra were obtained by atmospheric-pressure chemical ionization (APCI) in the positive mode, with the following conditions: capillary voltage: 4500 V; charging voltage: 2000 V; dry gas temperature: 200 ^o^C; APCI temperature: 350 ^o^C; mass range: 50-1600 m/z. The m/z value of each chromatogram peak was assigned according to the reported study^4^.

**Cell culture and cell study.** Hela cells were cultured on DMEM (Gibco) supplemented with 10% v/v FBS, 50 units/mL penicillin, and 50 μg/mL streptomycin. Cells were incubated in a humidified CO_2_ incubator (5% CO_2_) at 37 ^o^C. For MTT assay, western blot analysis and LC-MS^2^ proteomics, samples were prepared in four different groups of HeLa cells for comparison. (1) Light- / Ir-: The cells were cultured with neither light nor **B2**. (2) Light- / Ir+: The cells were treated with 0 to 32μM of the iridium complexes for MTT assay and 8μM of **B2** for western blot and proteomic sampling but without light. (3) Light+ / Ir-: The cells were exposed to 1 sun light for desired time but without any iridium complex being treated. (4) Light+ / Ir+: The cells were treated with same amount of iridium complex as (2) and same light as (3).

**Subcellular localization and phenotype imaging.** Hela cells (passage number <30) were grown on the coverglass-bottom confocal dish for 24h (coverglass size: 22x22x0.13 mm). **B2**, **B4**, **B1**, and **B3** of 5μM final concentration were treated to the cells and incubated for 2 hours. To monitor a localization pattern, 100μM of LysoTracker^TM^ Deep Red (Invitrogen, L12492) was co-treated for 0.5-1hour. After incubation, cell culture media was exchanged to fresh DMEM. The samples were imaged with Carl Zeiss LSM780NLO confocal laser scanning microscope (Jena, Germany) using 63X objective lens. The live cells were imaged in the incubator (37 °C, 5% CO_2_). 561nm laser was used to excite **B2**, **B4**, **B1**, and **B3** complexes. Emission was gained at 590-660nm for **B2** and**B1** and 660-740nm for **B4** and **B3.** LysoTracker^TM^ DR signal was gained using 633nm excitation pulse at 655-750nm detection range. To observe cell death phenotype and lysosomal membrane permeabilization via a probe fluorescence loss, Hela cells were seeded and grown on a confocal dish with DMEM in CO_2_ incubator (37 ^o^C, 5% CO_2_) for 24h. To stain mitochondria,Tom20-mApple Plasmids were transfected into grown cells on confocal dish with TurboFect® Transfection Reagent (Thermo Fisher, R0531). After 24h, 4μM of **B2** was treated to the cells to 1000:1 = DMEM: DMSO v/v ratio and incubated for 2 hours. 100μM LysoTracker^TM^ Deep Red probe was then treated and incubated for 0.5-1h. After washing and replacing the culture media, the live cells were recorded using confocal laser scanning microscopy. 633nm laser was used to excite LysoTracker^TM^ (emission collected at 703nm) and 561nm laser was used to excite mApple (emission at 585nm). 561nm excitation pulse was expected to sensitize **B2** simultaneously. Timelapse images were collected for 0 to 300 seconds (10 secs/frame) by auto-focusing at every three shots. The fluorescence intensities of LysoTracker^TM^ and mApple were evaluated based on the sum of PL at each corresponding channel. PL intensity at each frame was normalized by that of the first shot (t=0). Motility of lysosomes were tracked using Image J/Fiji software and its TrackMate plugin. The green lysosome content signal of LysoTracker^TM^ at t=0 was spotted as magenta, and its motion track was highlighted by a yellow line.

**Cell viability assay (MTT assay).** Hela cells were incubated with the supplemented DMEM in CO_2_ incubator. To evaluate cytotoxicity of the four iridium complexes, 3-(4,5-dimethyl-2-thiazolyl)-2,5-diphenyl-2H-tetrazolium bromide (MTT) assay was conducted. 1.5 x 10^5^ cells/mL of the cells were seeded on 96-well plate and incubated for 24h. 100μL of 0.0625 – 32μM of **B2**, **B4**, **B1**, and **B3** solution (DMSO: DMEM = 1:100) were treated to each well. After 2h incubation, the media was changed to 100μL of fresh DMEM and solar simulator was utilized for photosensitization (60 sec, 0.6 J/cm^2^). Extra overnight incubation (37 ^o^C, 5% CO_2_) in dark incubator followed. 25 μL of MTT solution (5 mg/mL PBS, pH 7.4) was then added to each well and the cells. After 3-hour incubation, the old media was carefully exchanged with 100μL solubilization buffer ([DMF (50% v/v), sodium dodecyl sulfate (10% w/v), 99.9% AcOH (0.4% v/v), and 1 N HCl (0.4% v/v)] and placed under dark room for 24h at room temperature. The absorbance of formazan was monitored at 570nm using SpectraMax M5 microplate reader (Molecular Devices, Sunnyvale, CA, USA). The acquired values were normalized by the average value of negative control (DMSO: DMEM = 1:100, without Ir complex, with or without light exposure) for each plate.

**Intracellular uptake of Ir complexes (ICP-OES).** Hela cells were cultured in a 60π dish until they reached 70~80% confluency. Following this, they were treated with a 5μM solution containing B1, B2, and C2 in DMEM for 2 hours. After the treatment, the cells underwent three gentle PBS washes and were detached using trypsin. The number of detached cells was counted in 1 ml of DMEM and transferred to a glass vial. Subsequently, 1mL of 65% HNO_3_ (TraceMetalTM Grade, Thermo Fisher) was added, and the mixture was heated at 90℃ for 1 hour. The resulting digested cell solutions were diluted with 4mL of LC-MS grade water (Merck) and analyzed using inductively coupled plasma-optical emission spectrometry (ICP-OES) (700-ES, Varian Inc.).The acquired amount of Ir complexes was then divided by the initial number of cells yielding uptake amount per cell.

**Western blot analysis of autophagic flux related proteins (LC3-I/II, P62).** Hela cells were grown with the supplemented DMEM under 37 ^o^C, 5% CO_2_ atmosphere on 6 well plate to >90% confluency. 8μM of **B2** in DMSO was treated (1000:1 = Cell culture media: DMSO) and incubated for 2 hours. After media exchange, light was irradiated with solar simulator (26.4mW/cm^2^, 90 sec). The cells were extracted using lysis buffer [RIPA protein extraction solution (50 mM Tris-HCl pH 7.5, 10mM NaCl, 1% NP-40, 0.5% deoxycholic acid, 0.1% SDS, 1mM PMSF), Halt^TM^ protease inhibitor cocktail (Thermo Fisher Scientific, MA, USA). The protein concentration of the lysates were quantified with bicinchoninic acid (BCA) assay (Thermo Fisher Scientific, MA, USA) before loading. With SDS-PAGE loading buffer (Biosesang, Gyeonggi-do, Korea), protein denaturation was done for 5 minutes at 90 ^o^C in heat block, followed by 5-minute cooling at 4 ^o^C. The denaturated solution was separated by SDS-PAGE gel electrophoresis. The separated proteins on the gel were then transferred to the nitrocellulose membrane (Pall corporation, NY, USA) and checked by Ponceou S (0.1% (w/v) Ponceau S in 5% acetic acid/water) staining. The stain was removed with 0.1% Tween-20 Tris-buffered saline (TBST) and blocked with 2% skim milk for 1 hour. The membrane was incubated with primary antibody in 2% skim milk for 1 hour. 1:1000 diluted SQSTM1 polyclonal antibody from rabbit (Thermo Fisher Scientific, USA) was used to probe P62 and 1:500 diluted anti-LC3-I/II polyclonal antibody from rabbit (Sigma Aldrich, USA) for LC3-I and II detection. Non-attached antibody was strictly washed with TBST. As secondary antibody, anti-rabbit HRP was incubated for 30 minutes, followed by extra TBST wash for three times. Western blot chemiluminescence signal was monitored by G:BOX Chemi XRQ (Syngene, Cambridge, UK) after development with Clarity reagent (Bio-rad, CA, USA). Intensities of the observed bands were quantified with Image J software.

**Quantification of western blot signal intensity.** Band intensity of western blot film was quantified with Image J software. ROI was selected to include the broadest band. The same ROI area was used for each independent experimental set. The signal intensity was measured using Image J measurement tool and expressed as (255-X_measurement_). Same was done for a background signal. The true band intensity was calculated as the subtract of the inverted band and the inverted background signal. For LC3-I/II, 17/14 kDa band signals were collected. For P62, >180 kDa and 55 kDa signals were gathered. The average signal intensities from hv-/Ir-, hv-/Ir+, and hv+/Ir- were taken as control.

**Flow cytometry analysis by** **fluorescence-activated single cell sorting.** 70-80% confluence of HeLa cells incubated in humidified 5% CO_2_ incubator at 37 ^o^C were treated with 8 μM B2 and B4-containing DMEM (1 vol. % DMSO) for 2 h and irradiated with solar simulator light (26.4mW/cm^2^, 60 sec, 1.584 J/cm^2^). After 6 h additional incubation, cells were harvested with 0.25% trypsin-EDTA (gibco) and washed twice with PBS. Cells were centrifuged and suspended in 100 μL 1X annexin-binding buffer (Cat. no. V13241, Invitrogen™). Then, cell suspensions were treated with 5 μL Alexa Fluor^®^ 488 annexin V and 1 μL 100 μg/mL propidium iodide (PI) working solution (5 μL of the 1 mg/mL PI stock solution in 45 μL 1X annexin-binding buffer) and incubated at room temperature for 15 min. After the incubation period, 400 μL of 1X annexin-binding buffer was added and cell suspensions were kept on ice. The stained cells were analyzed by flow cytometry, measuring the fluorescence emission at 527 nm (excitation 488 nm) for Alexa Fluor® 488-annexin V and 660 nm for PI.

**SNARE protein expression and purification** Proteins expressed and purified are human SNARE proteins, syntaxin-1A (STX1A; 191-268), synaptobrevin-2 (VAMP2; 1-97), and SNAP-25B, which were expressed in *E. coli* cells. The SNAP-25B is the full-length protein and the STX1A/VAMP2 contain SNARE motifs with C-terminal linker to allow the SNARE complex formation. The DNA sequence corresponding to the protein was cloned into pBT7-N-His vector (Bioneer) respectively, and then each recombinant plasmid was transferred into *E. coli* Rosetta (DE3) pLysS (Novagen). The cells were grown in Terrific Broth (TB) containing 100 μg/ml ampicillin and 24 μg/ml chloramphenicol at 37 ^o^C until OD_600_ reached to approximately 0.8. The overexpression of each protein was induced by the final concentration of 400 μM IPTG for 3 hours. The cells were then harvested by centrifugation (5700 rpm, 10 mins, 4 °C) and stored at -80 °C. The cell pellets were thawed and re-suspended in 50 ml of the lysis buffer. The lysis buffer for the STX1A was 20 mM HEPES (pH 7.2), 500 mM NaCl, 10% glycerol, 20 mM imidazole, 1 mM TCEP, 1 mM PMSF, 0.1% N-lauroylsarcosine sodium salt (N-lauroylsarcosine). The lysis buffer for VAMP2/SNAP-25B was 20 mM HEPES (pH 7.2), 100 mM NaCl, 10% glycerol, 20 mM imidazole, 1 mM TCEP, 1 mM PMSF. The re-suspension was lysed by Emulsiflex-C3 homogenizer (Avestin) at the pressure of 15,000~17,000 psi. Cell debris was removed by centrifugation (18,000 rpm, 30 mins, 4 °C) and the supernatant was incubated with Ni-NTA resin for 1 h at 4 °C. The resin was packed into a column and was washed with the washing buffer, the composition of which is same as the one of the lysis buffers without PMSF. STX1A was eluted with the elution buffer (20 mM HEPES (pH 7.2), 100 mM NaCl, 10% glycerol, 400 mM imidazole, 1 mM TCEP, 0.1% N-lauroylsarcosine). VAMP2 and SNAP-25B were eluted with the elution buffer without N-lauroylsarcosine. To corroborate the autophagy dysfunction by SNARE protein disturbance, each purified SNARE proteins was treated by 500 μM **B2** Ir complex in DMSO with light irradiation (26.4 mW/cm^2^, 30 mins) and then all the three proteins were mixed. After incubating the mixtures for 1 hour at room temperature, they were analyzed by 15% SDS-PAGE (STX1A: 11.9 kDa; VAMP2: 13.3 kDa; SNAP-25B: 26.0 kDa).

**Preparation of tryptic peptides for LC-MS/MS.** Hela cells were grown in 100 mm cell culture dish with the supplemented DMEM under 37 ^o^C, 5% CO_2_ environment. The cultured cells were incubated with **B2** for 2 hours and the culture media was exchanged with fresh DMEM. Then, the cells were irradiated with solar simulator light (26.4mW/cm^2^, 90 sec, 2.376 /cm^2^), followed by lysis using RIPA buffer (4 ^o^C, 20 min). Cell debris was eliminated from the lysate via centrifugation (16,000Xg, 10 min, 4 ^o^C). After denaturation at 95 ^o^C, the lysis solution was separated by SDS-PAGE gel electrophoresis. The protein loading quantity was controlled to carry on 50μg of protein for each sample based on BCA assay. Shot-Gun proteomics was used to analyze whole protein. Electrophoresis was done until the loaded protein lane reaches 1 cm length on SDS-PAGE gel and stained with Coomassie blue for 2 h. Coomassie blue was then removed with destaining solution (H_2_O: MeOH: AcOH = 5:4:1, v/v/v) for overnight. The destained gel was divided into 6 pieces by protein size and chopped up to 1 mm X 1 mm X 1mm cubes. The cubes in accordance with the six protein sizes were transferred to 1.5mL low binding microtubes (Eppendorf, Hamburg, Germany) and washed in the following steps: 1) 5 min with 150 μL TDI water on thermo-shaker for three times, 2) 5 min with 150μL 0.1 M ammonium bicarbonate (ABC) on the shaker for three times, 3) 5 min with 0.1 M ABC/ MeCN = 1:1 solution on the shaker for three times, 4) 5 min with MeCN on the shacker for three times, 5) The process from 2) to 4) was repeated 6) 20 min at 55 ^o^C with 500μL 100mM ABC: MeCN = 4:1 solution (700 rpm), and 7) repeat step 5) again. After removing the washing solvent, the samples were completely dried using a speed-vac. Reduction followed with the tubes filled with 150μL reaction solution (10mM dithiothreitol in 100mM ABC) being incubated on the thermo-shaker (800 rpm, 56 ^o^C, 1h). For alkylation, the solvent was than exchanged with 150μL of 55mM iodoacetamide in 100mM ABC solution and the tubes were incubated on the thermo-shaker in the dark (800 rpm, 25 ^o^C, 30 min). Using the shaker, washing steps followed as: 1) 5 min with 100mM ABC, 2) 5 min with 100mM ABC: MeCN = 1:1 solution, and 3) 5 min with MeCN. After repeating the previous 1)-3) washing step once again, the solvent was removed and dried using speed-vac. To the dried tubes, 25 ng/μL Trypsin Gold mass spectrometry grade (V5280; Promega, WI, USA) dissolved in 50mM ABC was added and incubated for overnight at 37 ^o^C. The tubes were vortexed, and the supernatant was transferred to new low binding microtubes. With 5% formic acid: MeCN = 2:1 solution, the remaining gels in the old microtubes were washed and transferred to the new tubes. The solutions were completely dried by speed-vac yielding peptide powder.

**Electron paramagnetic resonance (EPR) spectroscopy.** EPR spectroscopy was performed under room temperature using 4-hydroxy-2,2,6,6-tetramethylpiperidine (4-OH-TEMP) as a trap for ^1^O_2_ to yield 4-hydroxy-2,2,6,6-tetramethylpiperidine 1-oxyl (4-OH-TEMPO) free radical and 5,5-dimethyl-1-pyrroline *N-*oxide (DMPO) as a trap for hydroxyl radical to yield DMPO-OH and DMPO-OOH. The 1M stock solution of 4-OH-TEMP and DMPO in water were diluted either with pure water or the 10 mM stock solution of B4 in DMSO by 4:1 ratio (v/v) into 1.5 ml microtube. The diluted mixture was then treated with or without 530 nm LED for 3 minutes (1 J/min·cm^2^) and transferred to an EPR capillary tube for EPR spectroscopy.

**Tumor modeling and in vivo assessment of antitumor effect.** The animal experiments were conducted using the approved protocol (UNISTIACUC-20-11). To construct tumor model resistant to chemotherapy, Panc-1 cells were selected. 1.2 × 10^7^ Panc-1 cells in 80 μL PBS were subcutaneously inoculated at the flank of BALB/c nude mice (6 w, 16-18 g). PBS or drugs were administered as the tumor volume reaching around 50 mm^3^. To test chemo-resistant character of the tumor, 50 mg/kg or 100 mg/kg of gemcitabine were treated four times at the 1^st^, 4^th^, 8^th^, and 11^th^ day of the treatment. The mice were monitored for extra two weeks and euthanized at day 25. To assess the photodynamic efficacy of B4 compared to non-treated and non-irradiative condition, 80 μL PBS, or 10 mg/kg of B4 in 2.5% Kolliphor HS15 (Sigma-Aldrich, 42966) were injected intravenously on tail vein. For photodynamic therapy group, the tumor was irradiated with 635 nm laser (170 J/cm^2^) at 3 h post-injection of B4. The photodynamic therapy was repeated once again at day 4, and monitored until day 30. The mice were euthanized for dissection at the last day.

**Liver and kidney toxicity evaluation.** After 1 week of adaptation, BALB/c nude mice (8 w, 20 g) were injected with 100 µL of 1 mg/ml solution for twice a week. Total 2 weeks of treatment (4 injections) administered 10 mg/kg of B4. Extra 1 week without any treatment was given after the last administration to assure recovery before blood sampling via cardiac puncture. The serum was collected for the toxicity test based on representative markers: blood urea nitrogen (BUN), creatinine, alkaline phosphatase (ALP), alanine transaminase (ALT), and aspartate aminotransferase (AST).

**Microscopic histochemistry.** Panc-1 tumor samples were collected 24h-post treatment of the first administration of 100 mg/kg gemcitabine or 10 mg/kg B4 with 635 nm light irradiation. The samples for H&E staining, TUNEL assay, and immunofluorescence imaging of LC3 and p62/SQSTM1 expression were stored in 10% neutral buffered formalin (10% NBF). The stored samples were deparaffinized rehydrated through sequential dipping in xylene and 100%–70% gradient aqueous ethanol solution, and washing with tap water. To perform H&E staining, tissues were stained with hematoxylin (YD Diagnostics Corp., S2-5) and Eosin (Duksan, c73521), followed by dehydration with ethanol and xylene. For TUNEL stain, TUNEL Assay Kit-HRP-DAB (abcam, ab206386) was used according to the given procedure. Micromount (Leica, 3801730) was used as a mounting medium and the images were captured by virtual microscopy equipped with Olympus upright microscope B x 51. To visualize expression level of LC3 and p62/SQSTM1, tissues were unmasked with citrate buffer, washed, and blocked with 5% normal goat serum in TBST. LC3A/B-Alexa Fluor 488 conjugate (Cell Signaling Technology, 13082), SQSTM1 polyclonal antibody (Invitrogen, PA5-27247), and Goat anti-Rabbit Alexa Fluor^TM^ Plus 488 secondary antibody (Invitrogen, A32732) were used and further stained and mounted using VECTASHIELD Antifade Mounting Medium with DAPI (Vector Laboratories, H-1200-10). The immunofluorescent samples were imaged with Carl Zeiss LSM780NLO confocal laser scanning microscope (Jena, Germany) using 63X objective lens.

**2. Supporting figures**

**Figure S1.** Synthetic pathways toward the neutral iridium complex photosensitizers, **B2** and **B4**.

**Figure S2.** Synthetic pathways toward the cationic iridium complex photosensitizers, **C2**.

**
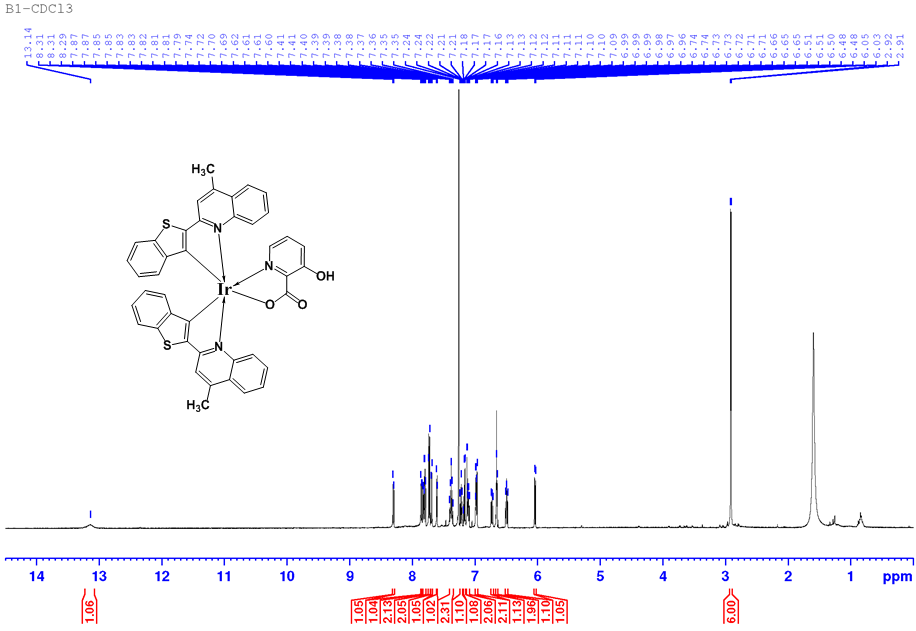
**

**Figure S3.** ^1^H-NMR spectrum of bis[2-(benzo[b]thiophen-2-yl)-4-methylquinoline]iridium(III) (3-hydroxy picolinate) (**B1**).

**
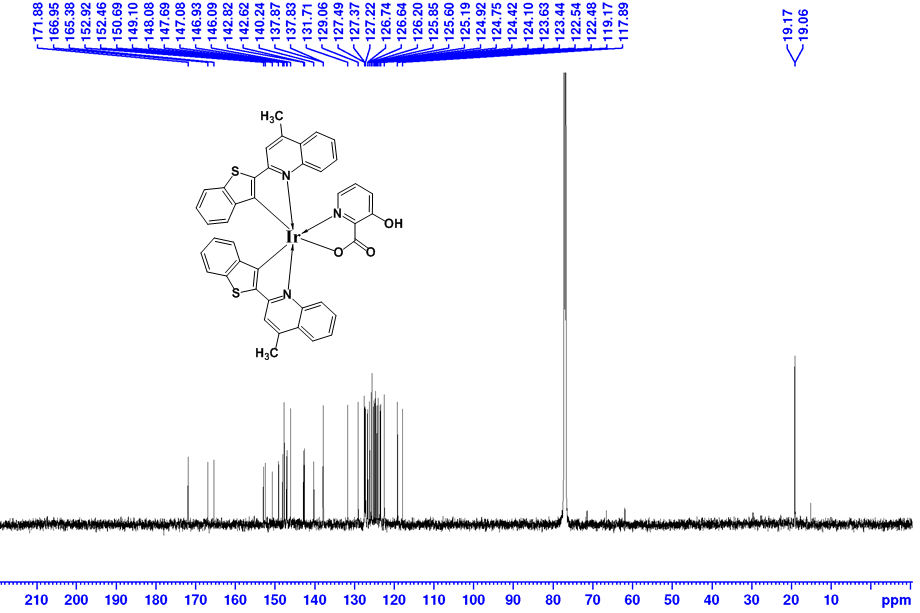
**

**Figure S4.** ^13^C-NMR spectrum of **B1**.

**
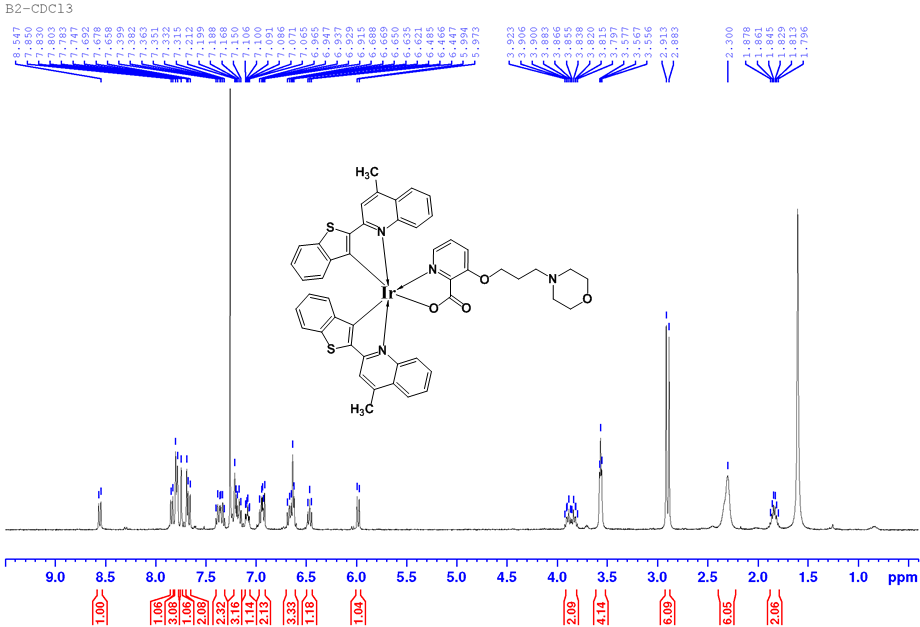
**

**Figure S5.** ^1^H-NMR spectrum of bis[2-(benzo[b]thiophen-2-yl)-4-methylquinoline]iridium(III) [3-(4-morpholinyl)propoxy]picolinate (**B2**).

**
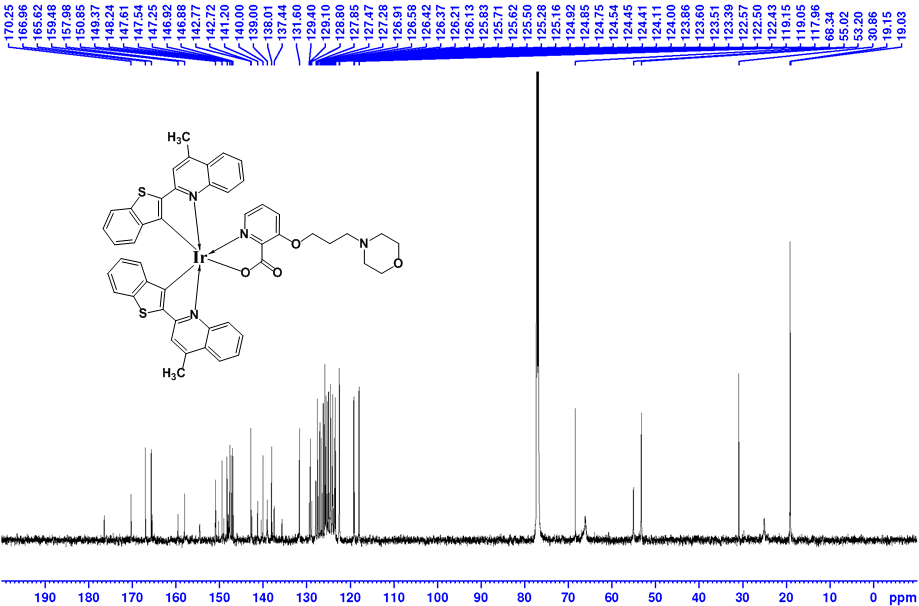
**

**Figure S6.** ^13^C-NMR spectrum of **B2**.


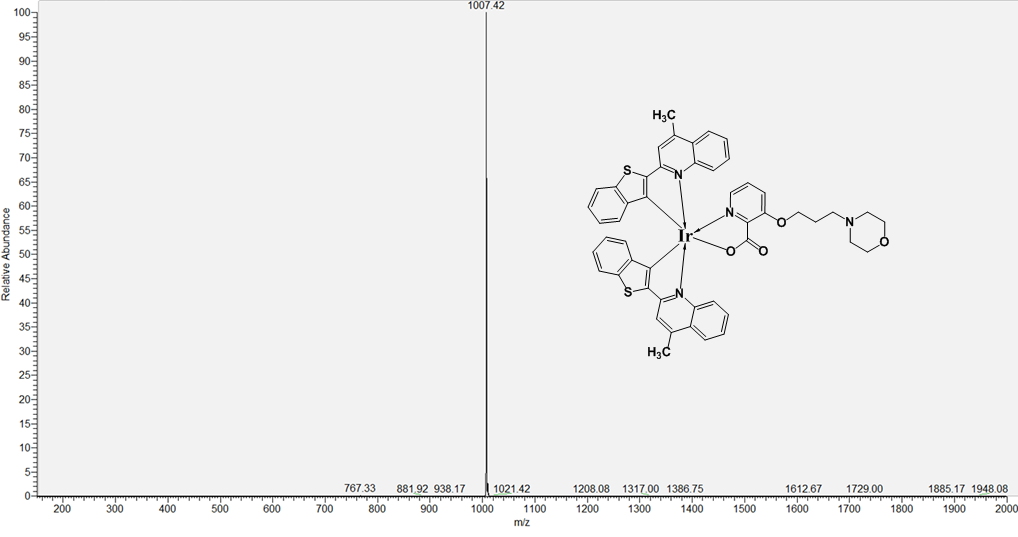


**Figure S7.** ESI-MS spectrum of **B2**.
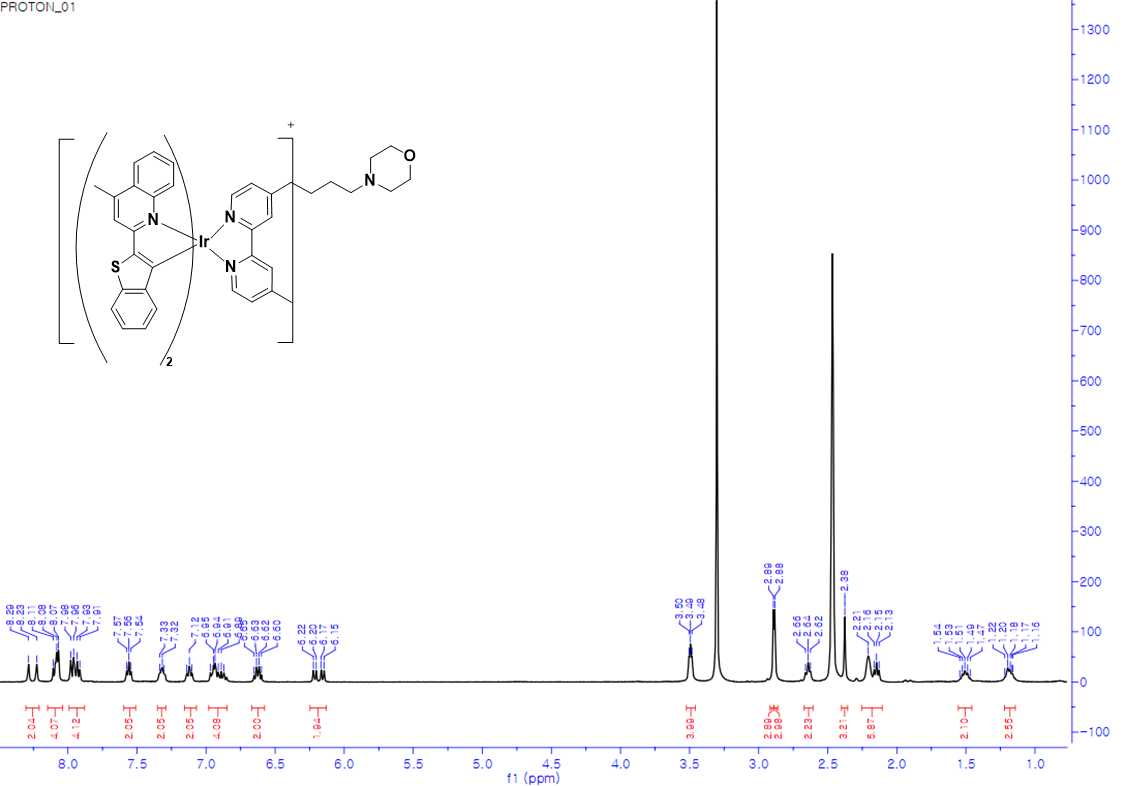


**Figure S8.** ^1^H-NMR spectrum of bis[2-(benzo[b]thiophen-2-yl)-4-methylquinoline]iridium(III)[ 4-(4-(4'-methyl-[2,2'-bipyridin]-4-yl)butyl)morpholine] (**C2**).


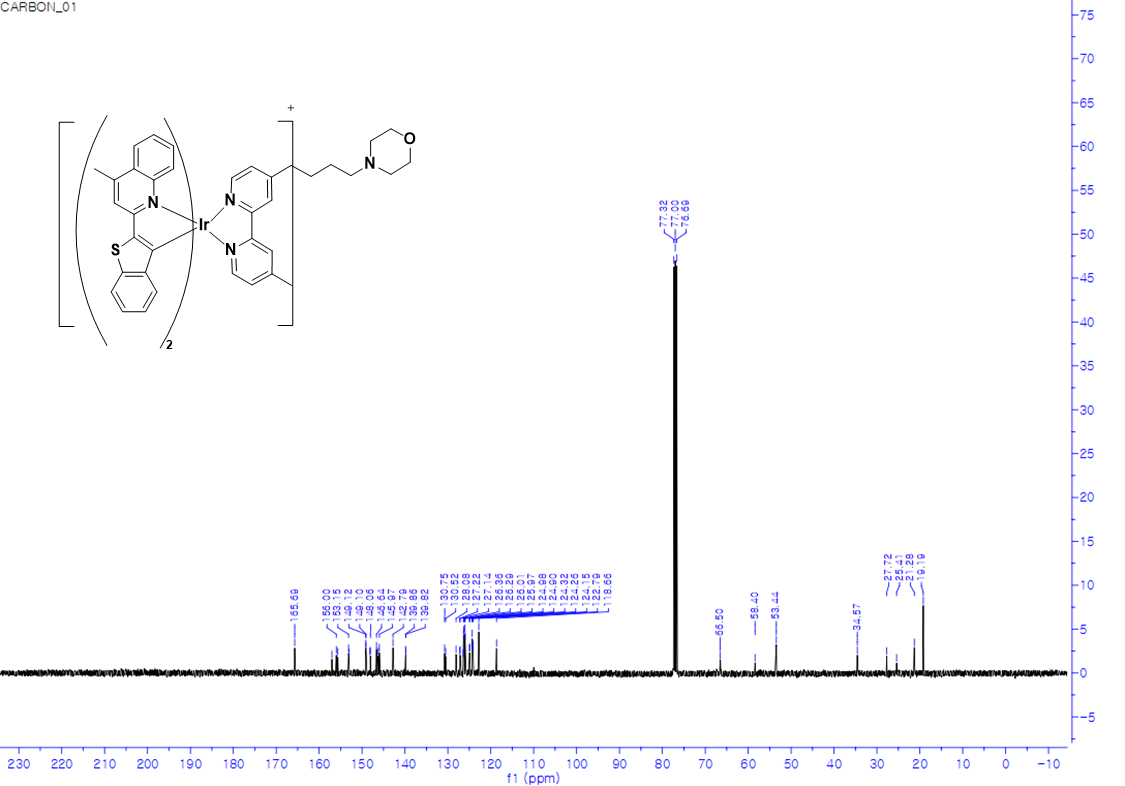


**Figure S9.** ^13^C-NMR spectrum of **C2**.


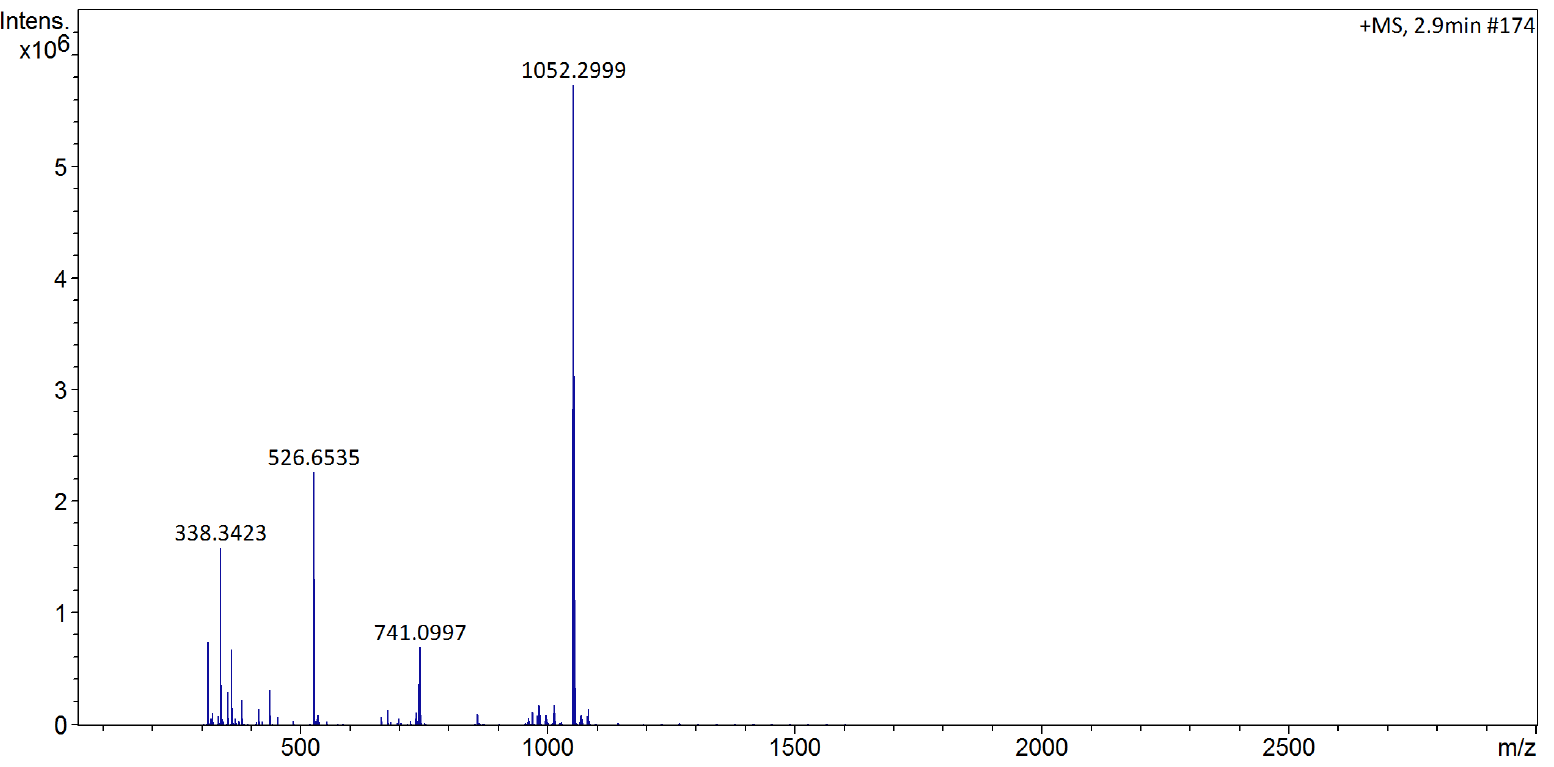


**Figure S10**. ESI-MS spectrum of **C2**.


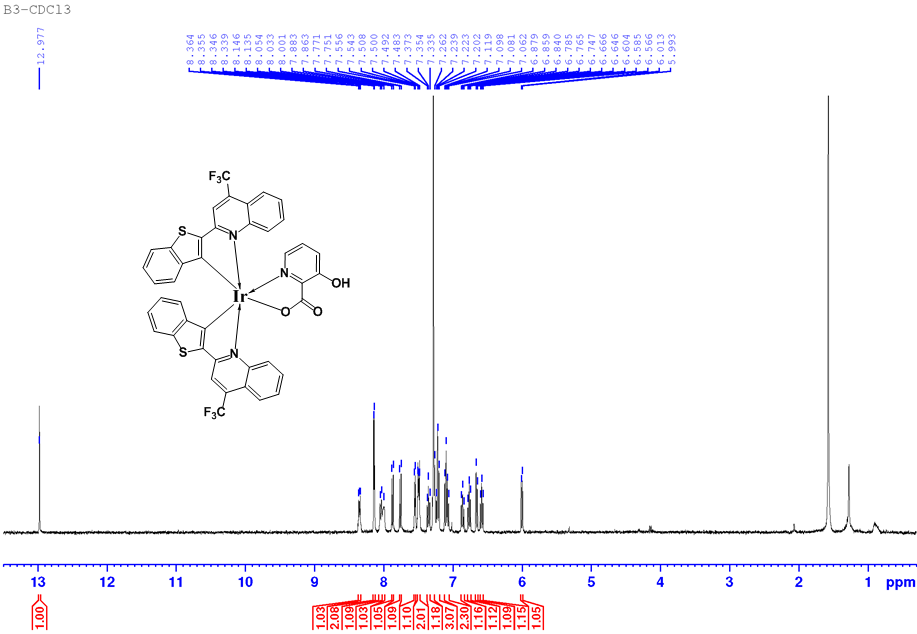


**Figure S11.** ^1^H-NMR spectrum of bis[2-(benzo[b]thiophen-2-yl)-4-(trifluoromethyl)quinoline]iridium(III) (3-hydroxy picolinate) (**B3**).


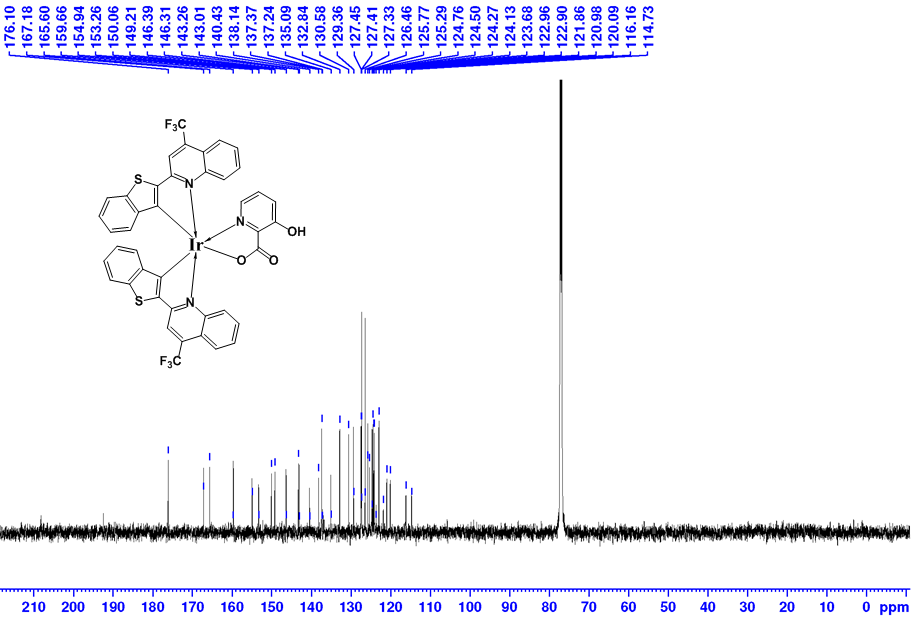


**Figure S12.** ^13^C-NMR spectrum of **B3**.


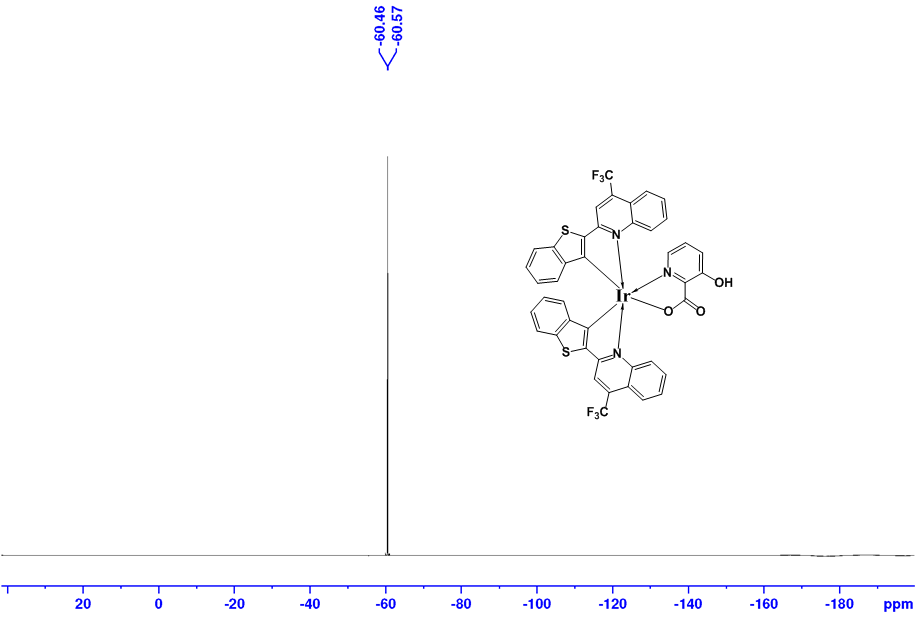


**Figure S13.** ^19^F-NMR spectrum of **B3**.


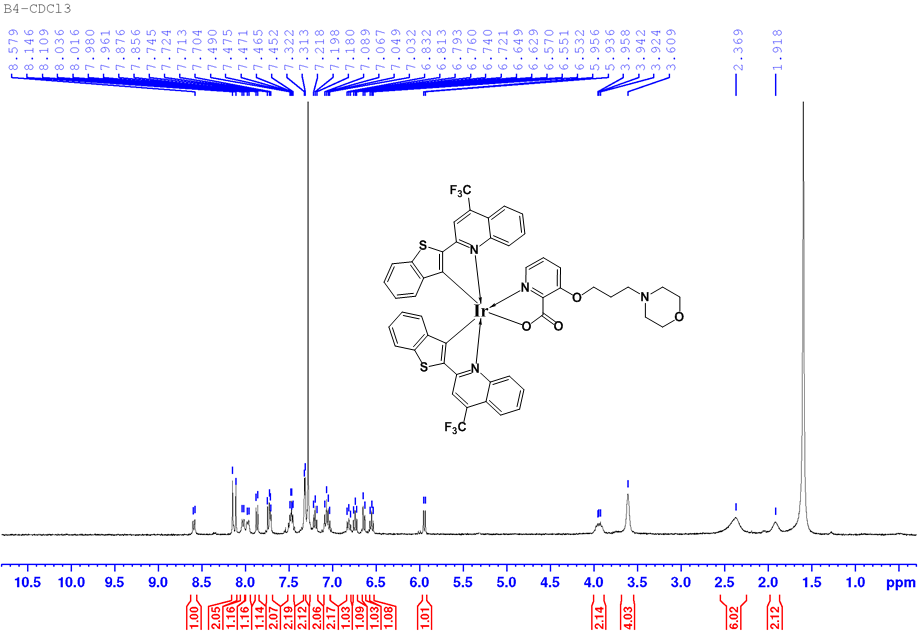


**Figure S14.** ^1^H-NMR spectrum of bis[2-(benzo[b]thiophen-2-yl)-4-(trifluoromethyl)quinoline]iridium(III) [3-(4-morpholinyl)propoxy]picolinate (**B4**).


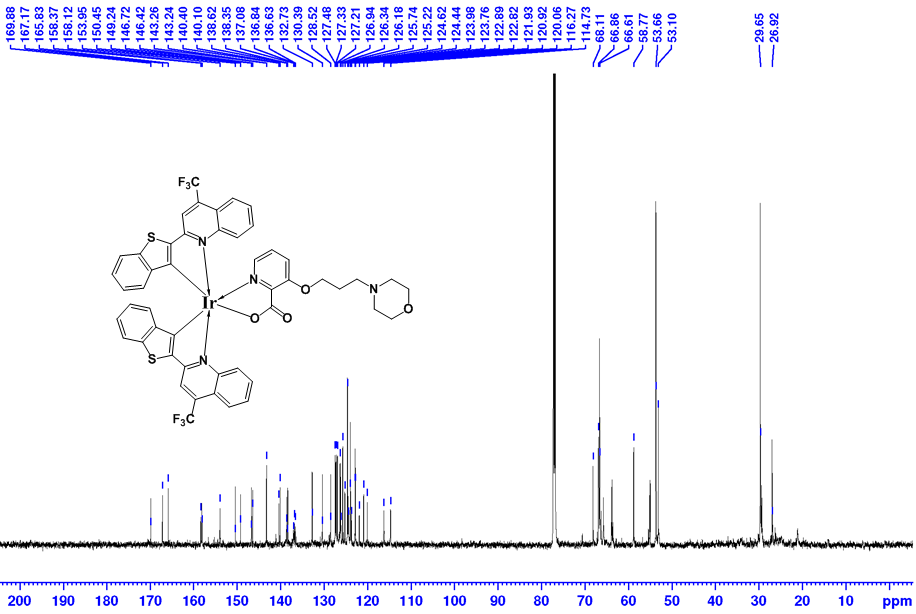


**Figure S15.** ^13^C-NMR spectrum of **B4**.


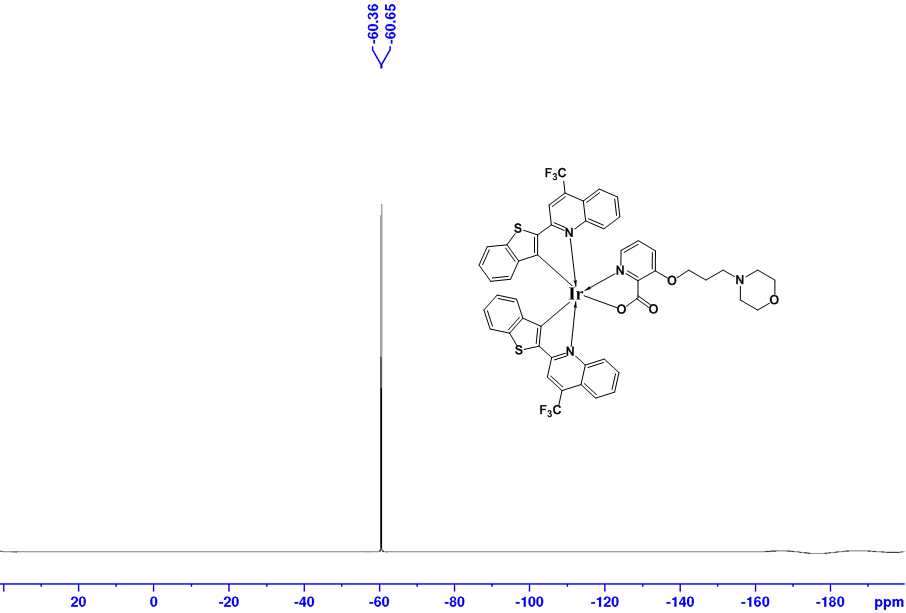


**Figure S16.** ^19^F-NMR spectrum of **B4**.


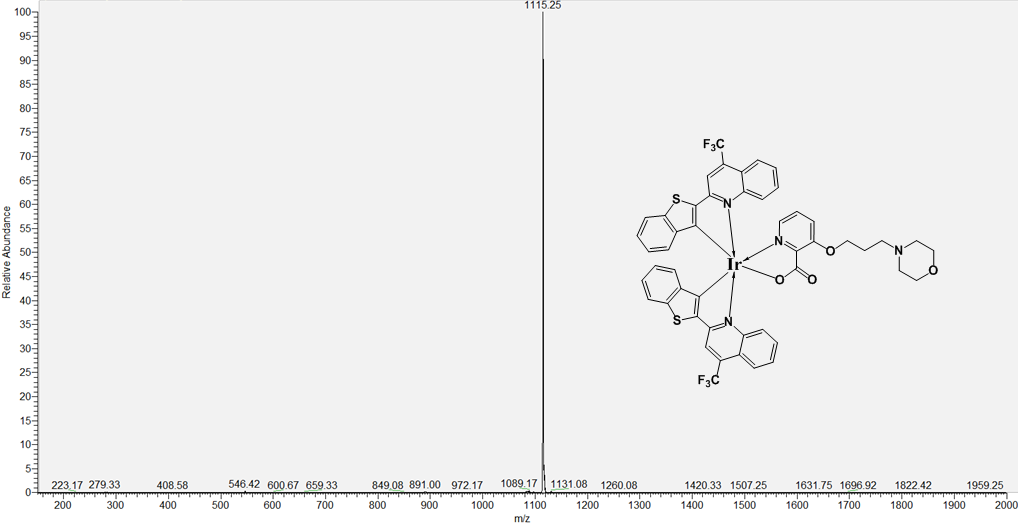


**Figure S17.** ESI-MS spectrum of of **B4**.


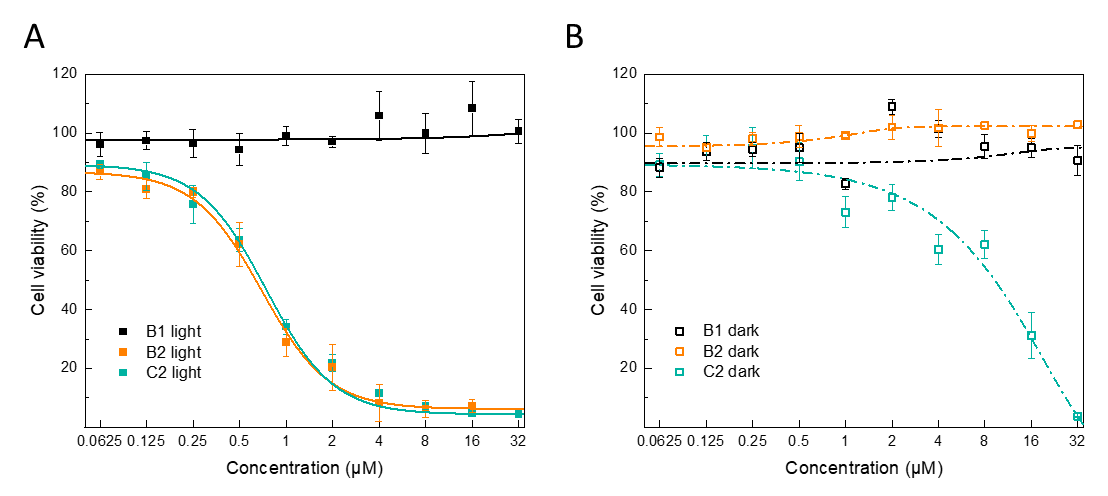


**Figure S18.** **Phototoxicity and biocompatibility of B1, B2, and C2 assessed by MTT assay** (a) with or (b) without photoexcitation.

**Figure S19.** **Dark toxicity of B2 and B4 at high concentration.** Dark toxicity of B2 and B4 at high concentrations were evaluated by MTT assay with HeLa cells.

**

**

**Figure S20.** **Cellular uptake amount of B1, B2, and C2 measured by ICP-OES.** Data are triplicated and shown with standard deviation.

**Figure S21.** **Absorption (solid line) and normalised photoluminescence (dashed line) spectra of B2 (red) and B4 (blue) in CH_2_Cl_2_.** B2 and B4 exhibited strong spin-allowed metal-to-ligand charge transfer transition (MLCT) bands at 522 nm and 574 nm, respectively. Emission spectra were recorded in deaerated dichloromethane solution at room temperature, after excitation at 480 nm for B2 and at 530 nm for B4. B2 showed red emission (650 nm), while B4 exhibited red-shifted near infra-red emission (715 nm) because of the strong electron withdrawing effect of –CF3.


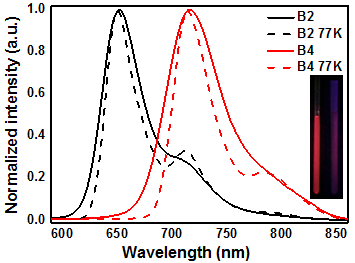


**Figure S22.** **Low temperature** p**hotoluminescence spectra of B2 and B4.** Photoluminescence spectra of B2 and B4 in CH_2_Cl_2_ solutions at 300K and frozen 2-methyl tetrahydrofuran solution at 77K (c = 2×10^-5^ M, solid line: fluorescence at 300K, dashed line: phosphorescence at 77K).

**Figure S23.** **Cyclic voltammogram of B2 and B4.** Oxidation and reduction potentials of B2 (red) and B4 (blue) were measured using cyclic voltammetry (ferrocene standard: 0.662 eV)


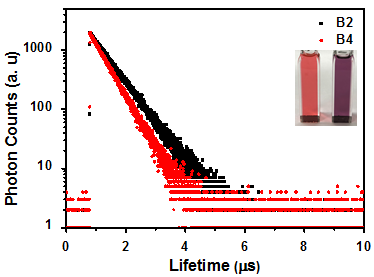


**Figure S24. Lifetime decay profiles of B2 and B4.** Comparison of lifetime decay profiles of B2 and B4 in degassed CH_2_Cl_2_ solution at 300K (c = 2×10^-5^ M).


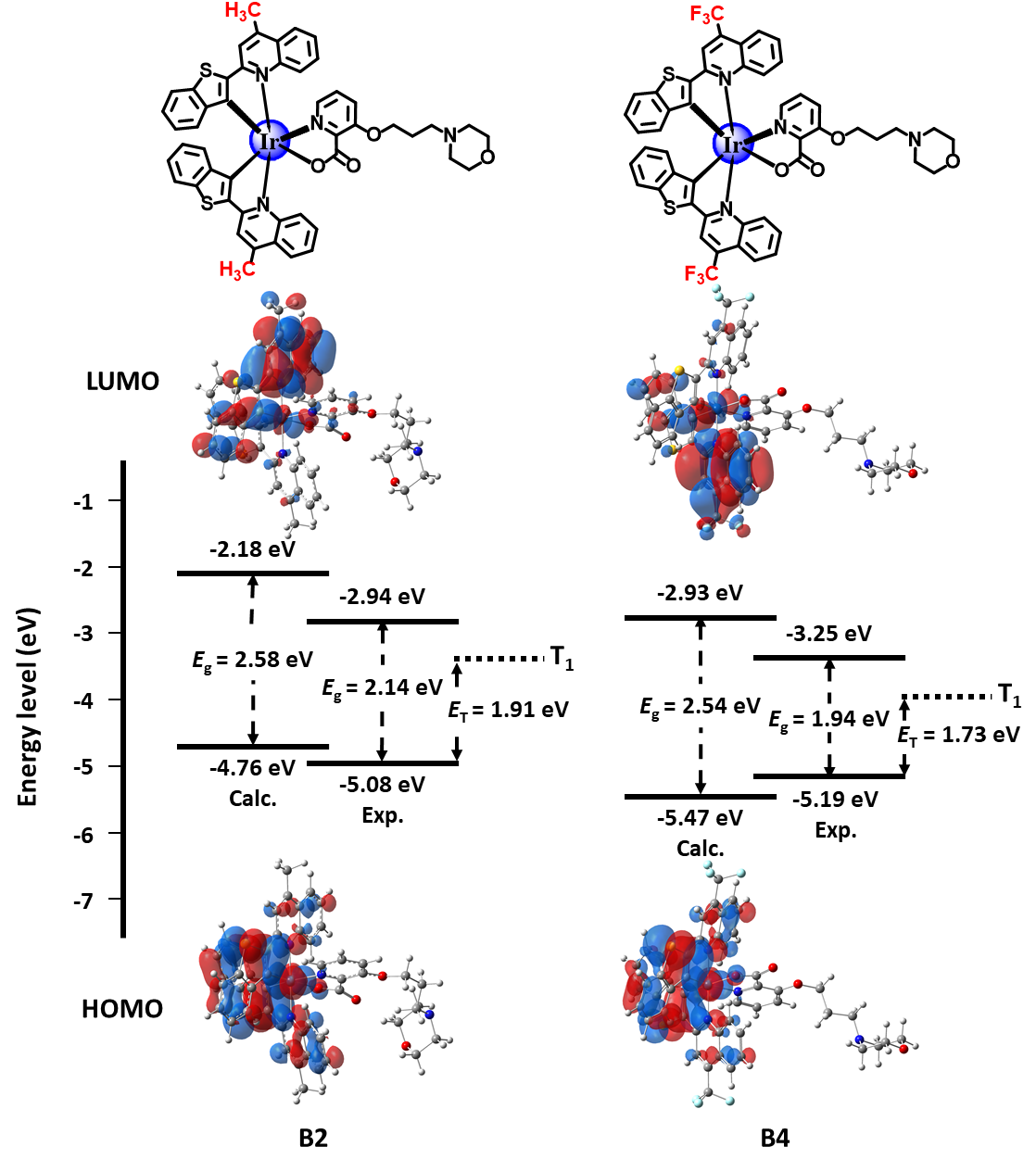


**Figure S25. HOMO and LUMO distributions, calculated energy levels from calculations and experimental energy level diagram of B2 and B4**. The experimentally acquired energy levels of the Ir(III) complexes were compared with density functional theory calculations and are shown with orbital distribution


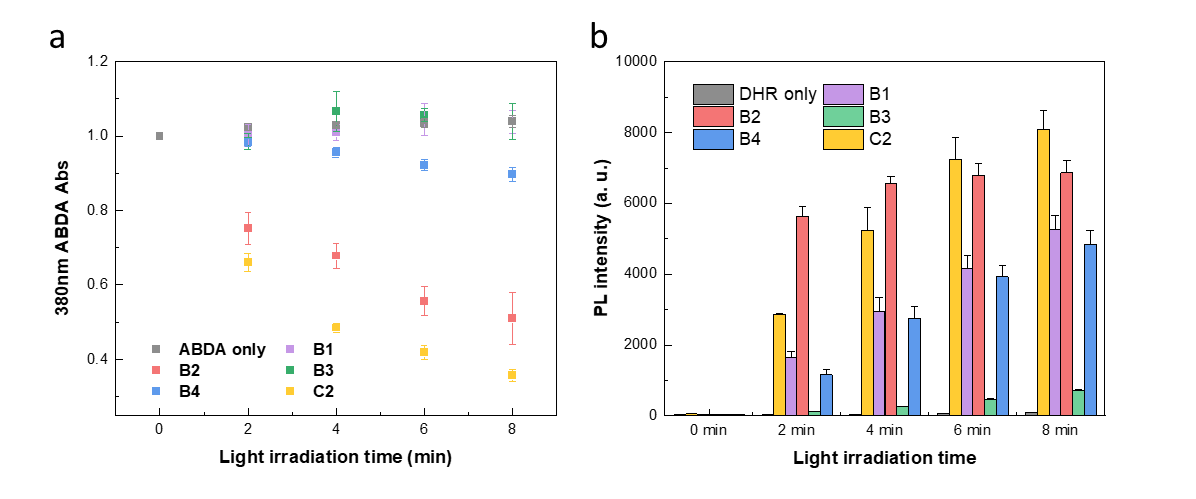


**Figure S26.** **ROS assay result of the Ir(III) complexes.** (a) ABDA absorbance decay by singlet oxygen was observed at 380 nm, which refers to type II ROS. (b) DHR123 assay result. Emission was collected at 525nm after 488nm excitation and the PL increase by time refers to type I ROS.

**
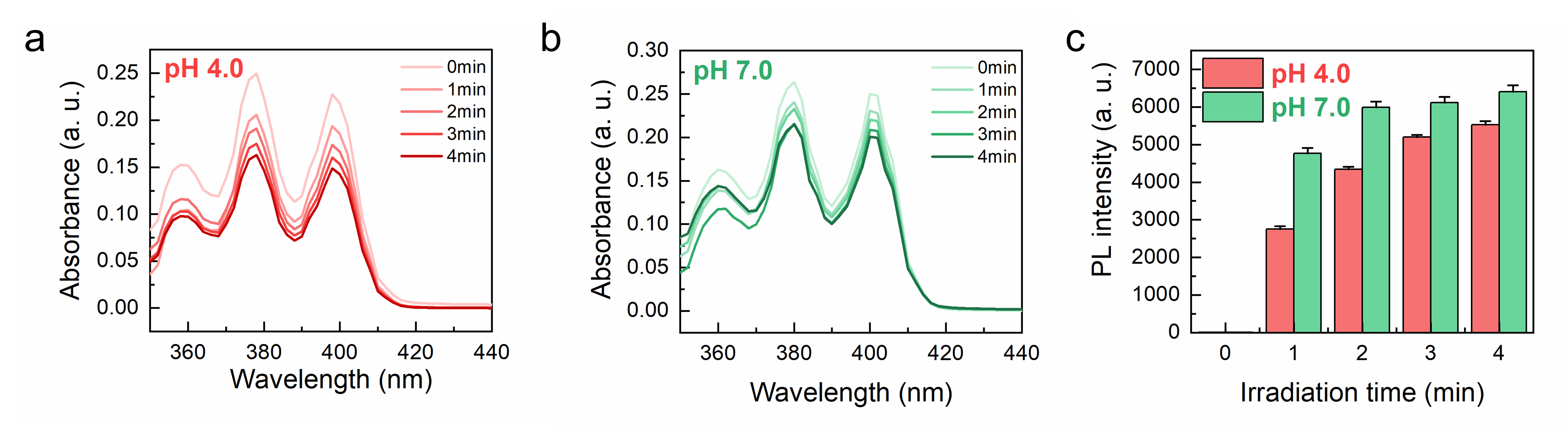
**

**Figure S27.** **ROS generation ability of B2 under acidic environment.** (a) ABDA absorbance decay was observed in pH 4.0 or (b) in pH 7.0 phosphate buffer solution. (c) DHR123 assay result. Emission was collected in pH 4.0 (red) or pH 7.0 (green) phosphate buffer solution.


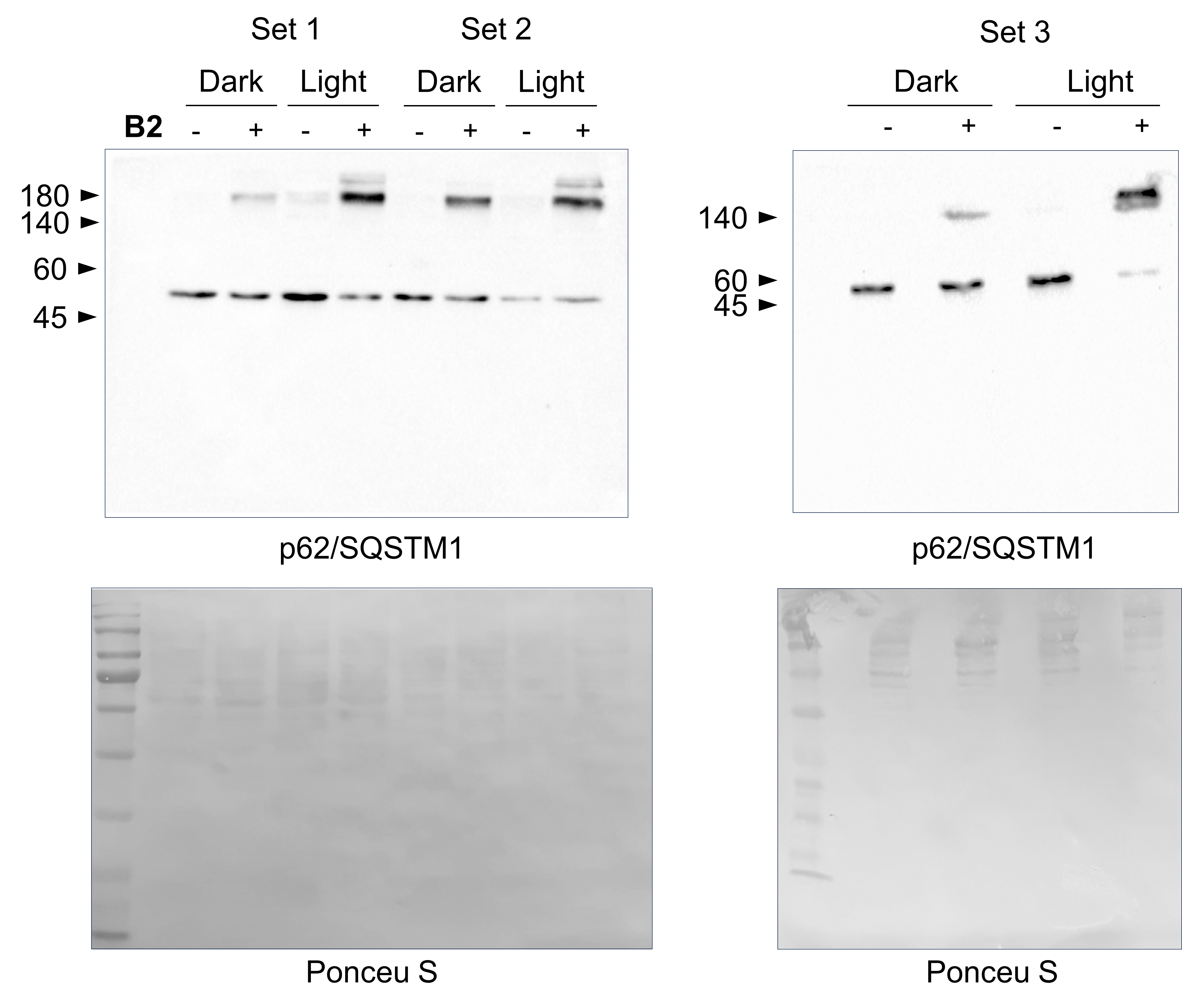


**Figure S28.** **Whole western blot images of p62.** Full-length blot of p62 shown with its corresponding Ponceu S staining. Experiments were conducted in triplicate with biologically independent samples.


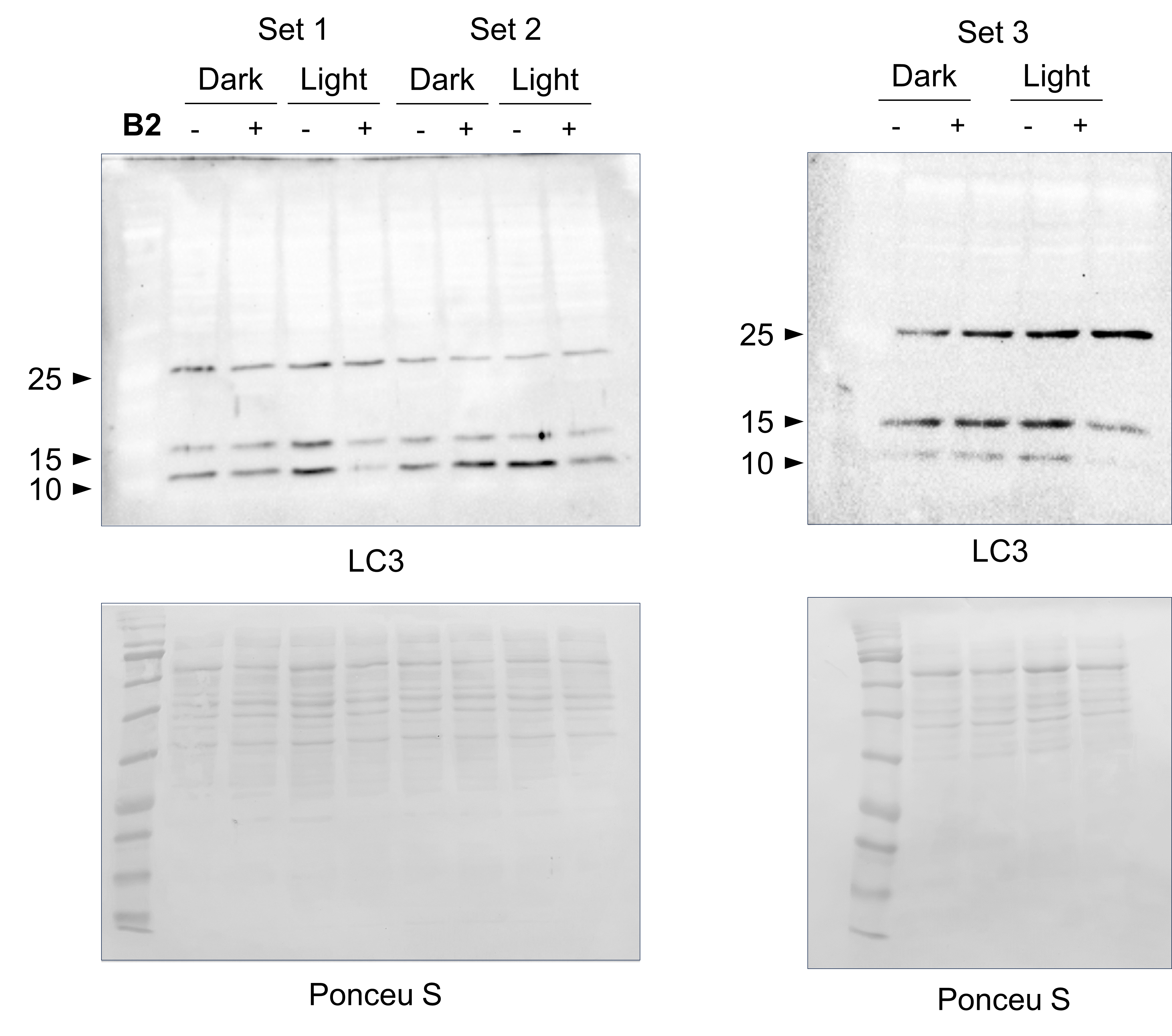


**Figure S29.** **Whole western blot images of LC3.** Full-length blot of LC3 shown with its corresponding Ponceu S staining. Experiments were conducted in triplicate with biologically independent samples.

**
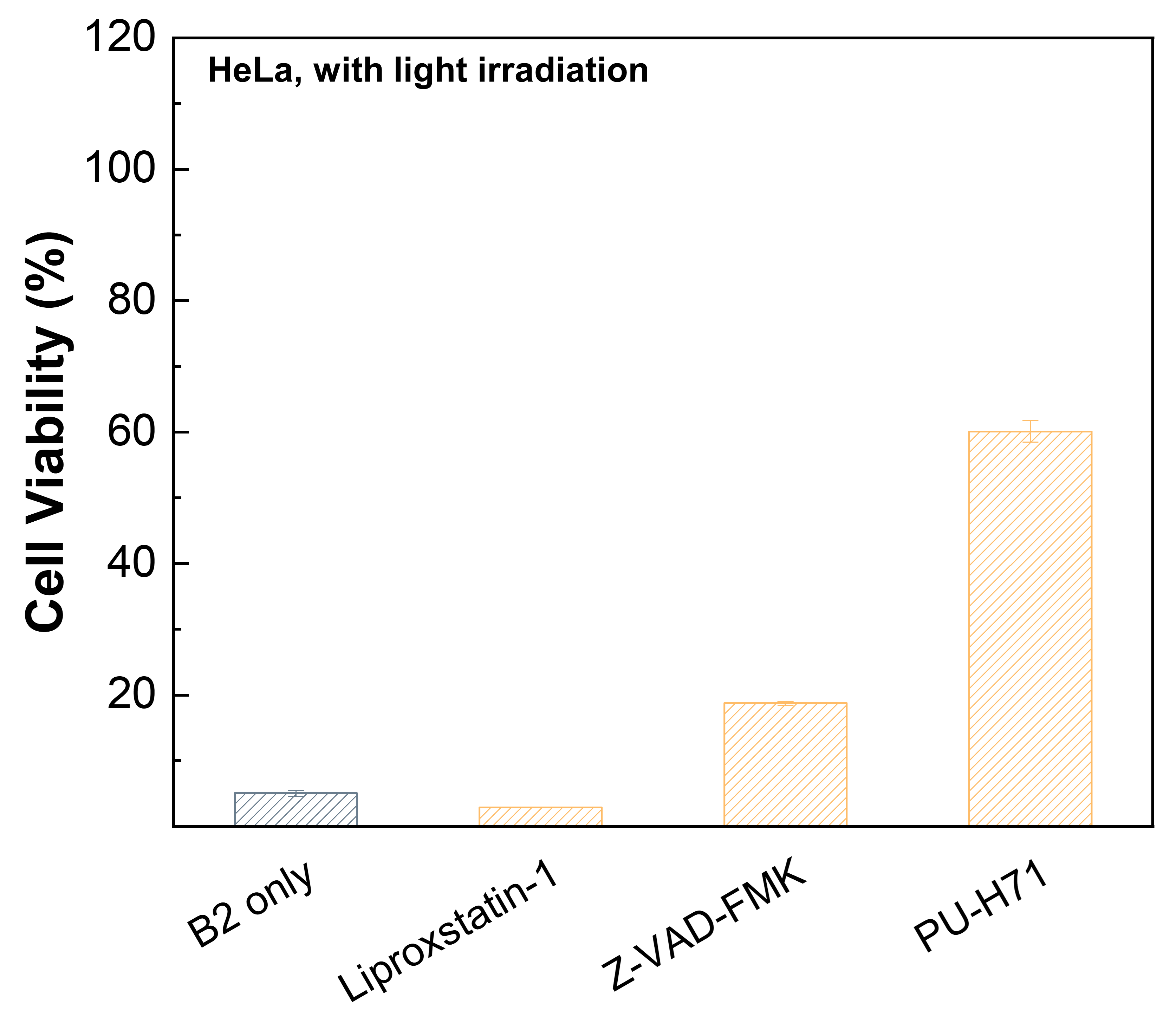
**

**Figure S30.** **Phototoxicity of B2 with cotreatment of inhibitors.** MTT assay conducted using B2 in HeLa cells, with or without inhibitors. 10 µM B2 in DMSO was treated and incubated for 2 h before being irradiated with 0.9 J of white light. 10 µM Liproxstatin-1 in DMF or 4 µM Z-VAD-FMK in DMSO was pre-treated for 16 h before B2 treatment. 1 µM of PU-H71 in DMSO was co-incubated with B2 for 2h. Data are presented as mean ± S.E.M (n = 4).


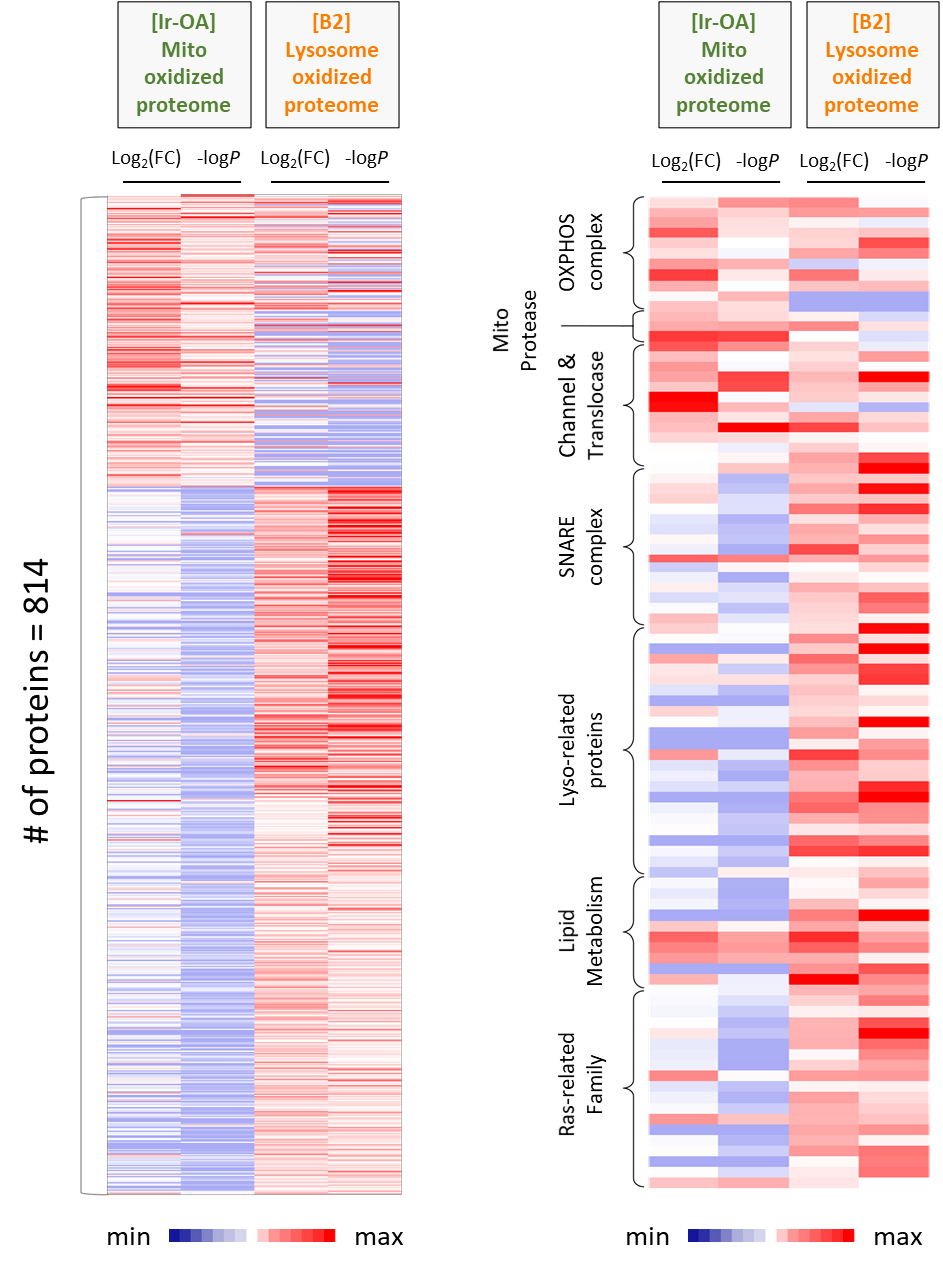


**Figure S31.** **Heat map of oxidized protein network of Ir-OA versus B2.** Highly oxidized proteins and low p-values were colored blue. Ir-OA proteome was retrieved from our previous publication.


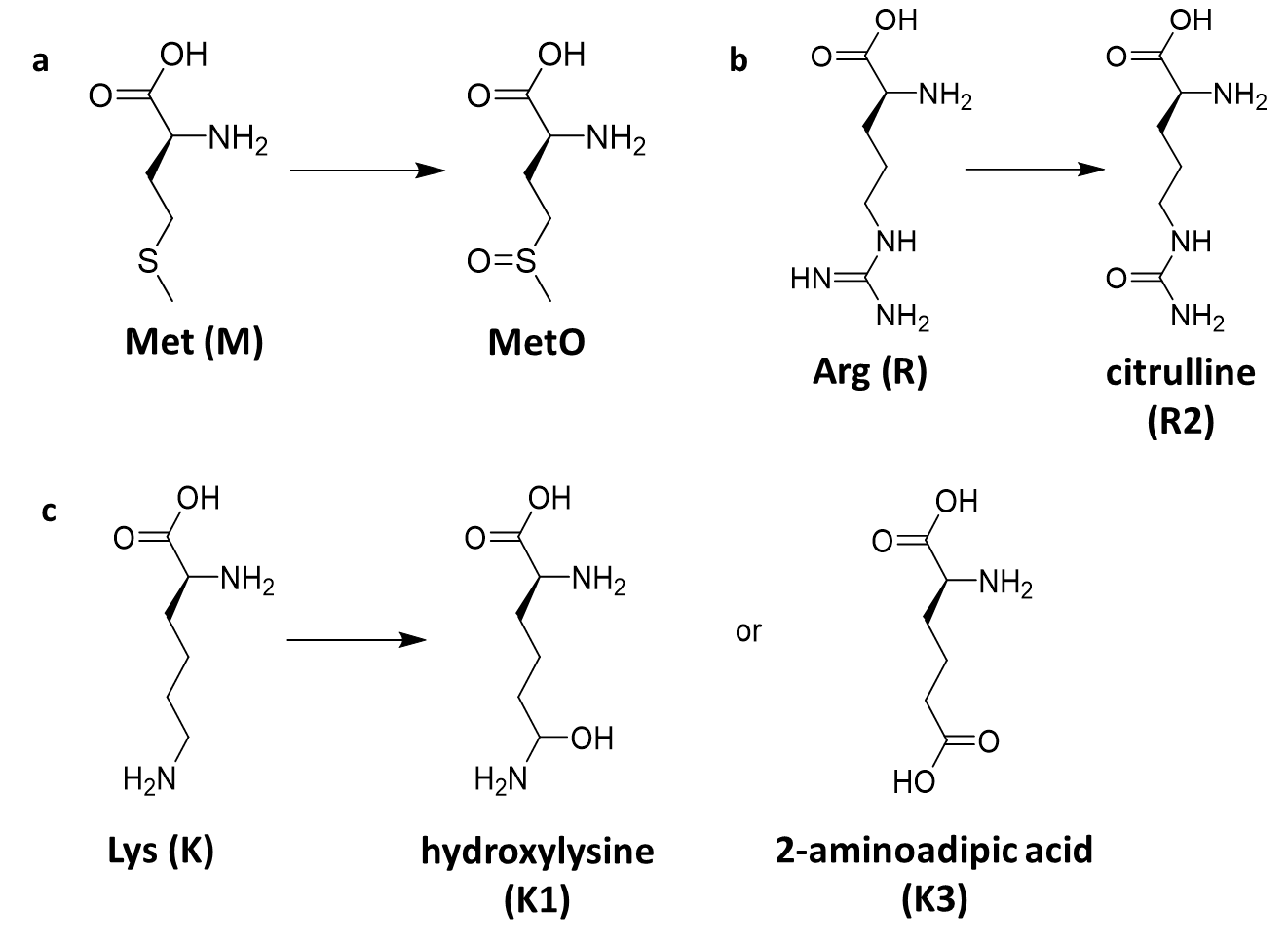


**Figure S32.** **Oxidative post-translational modifications that were covered in this report.** Ox-PTMs that were not detected by LC-MS/MS are not presented here.

**
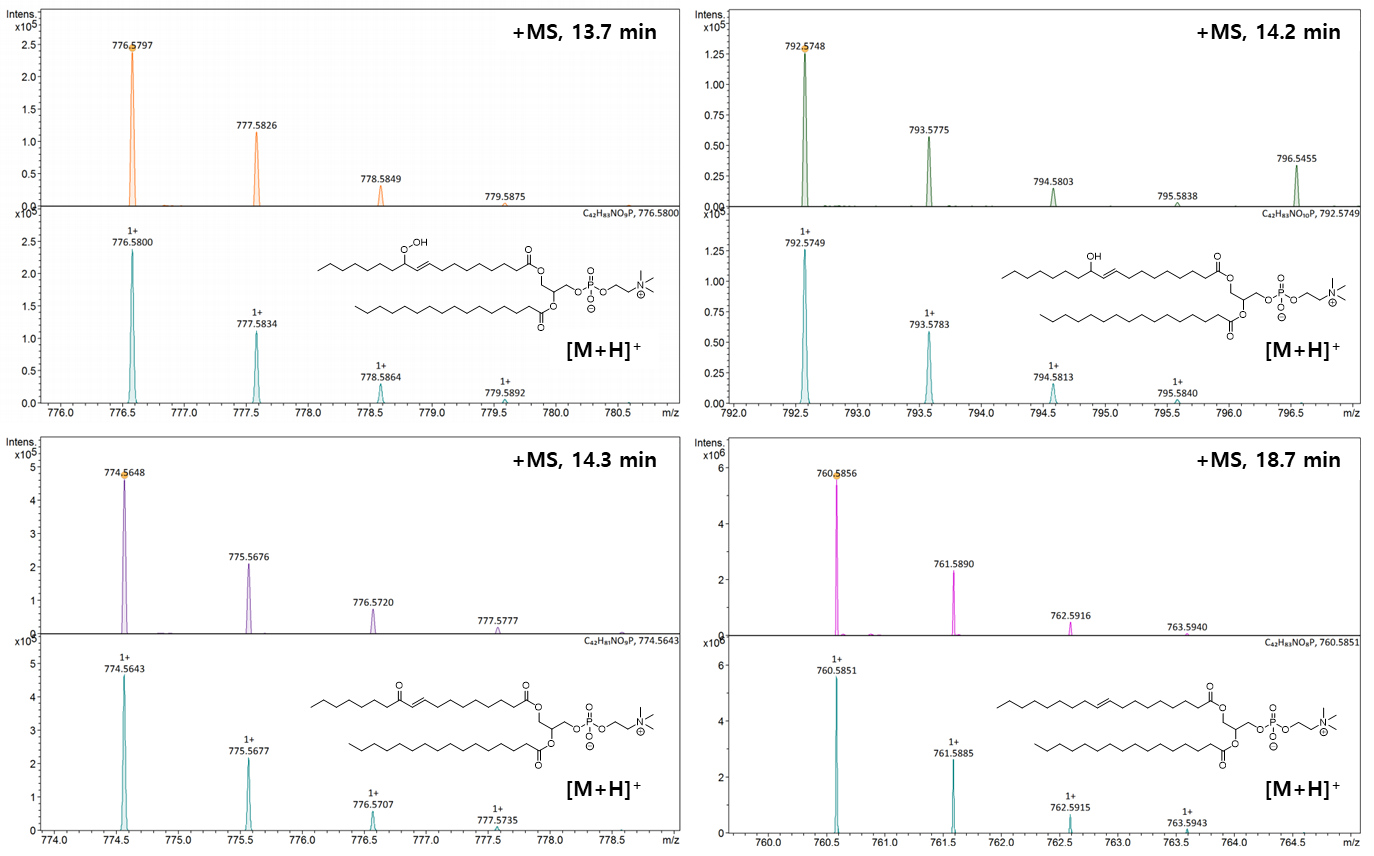
**

**Figure S33. HRMS analysis of phosphatidylcholine (PC) oxidation products at peak retention time (t_R_).** Calculated spectra are described bottom. Each peak retention time of chromatogram corresponds to PC oxidation products: PC-peroxide (t_R_ = 13.7 min), PC-alcohol (t_R_ = 14.2 min), PC-ketone (t_R_ = 14.3 min), and PC (t_R_ = 18.7 min).

**
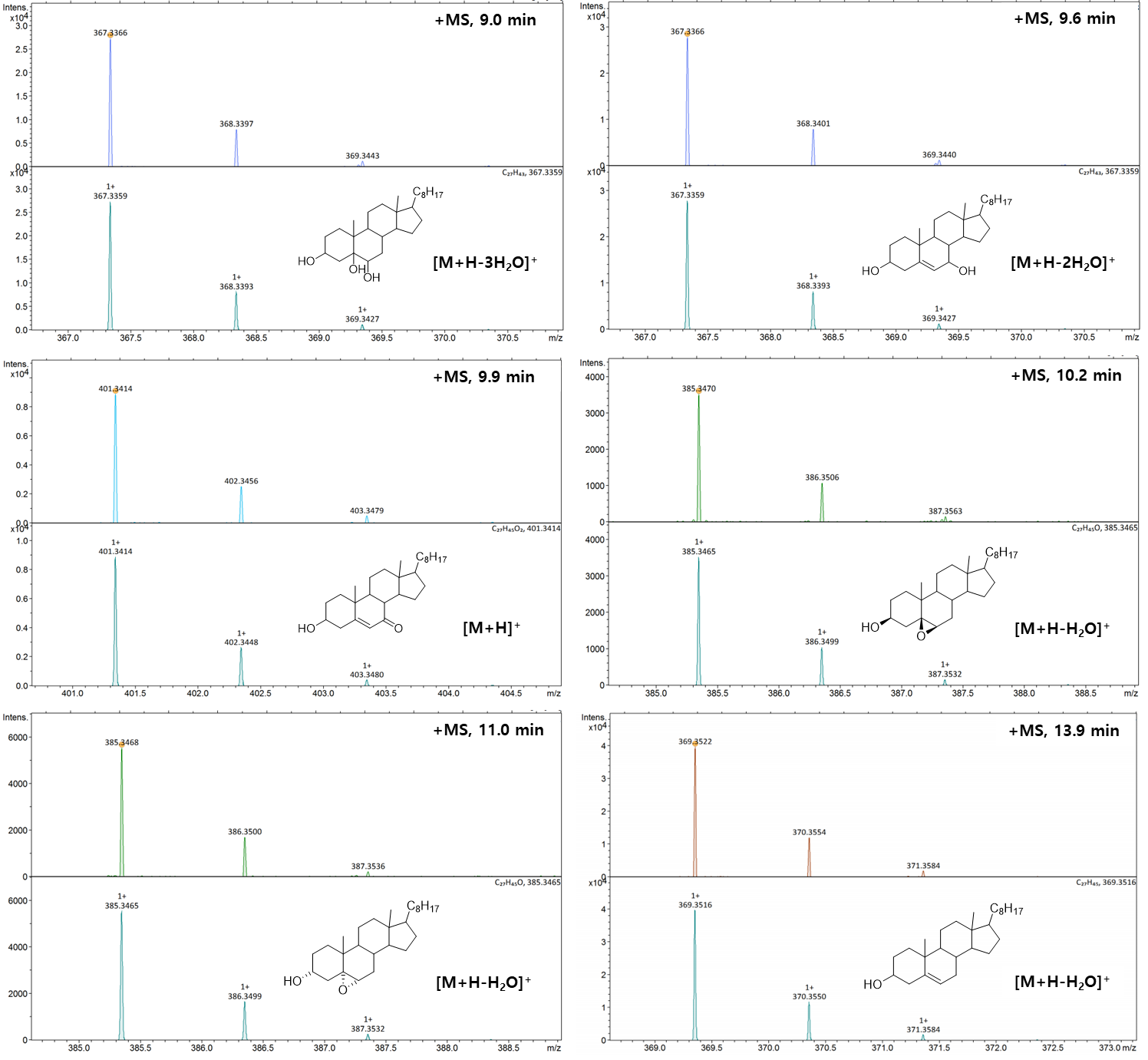
**

**Figure S34.** **HRMS analysis of cholesterol (Ch) oxidation products at peak retention time.** Calculated spectra are described bottom. Each peak retention time of chromatogram corresponds to Ch oxidation products: Triol-Ch (t_R_ = 9.0 min), 7-hydroxy-Ch (t_R_ = 9.6 min), 7-keto-Ch (t_R_ = 9.9 min), Ch-5α,6α-epoxide (t_R_ = 10.2 min), Ch-5β,6β-epoxide (t_R_ = 11.0 min), and Ch (t_R_ = 13.9 min).

**
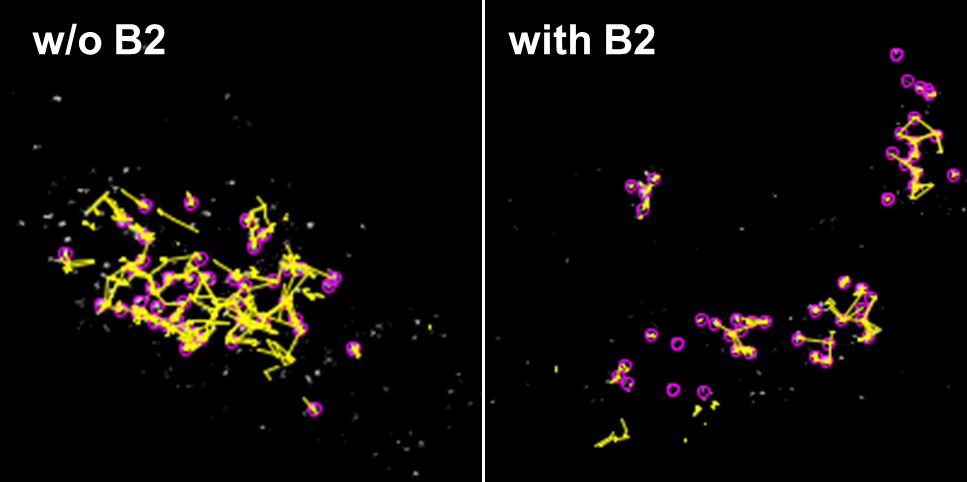
**

**Figure S35.** **Motility of lysosome with or without photosensitization.** Motility of lysosome with or without photosensitization was tracked using TrackMate. Magenta: initial position; yellow line: motion trace. t= 0~300 sec.

**
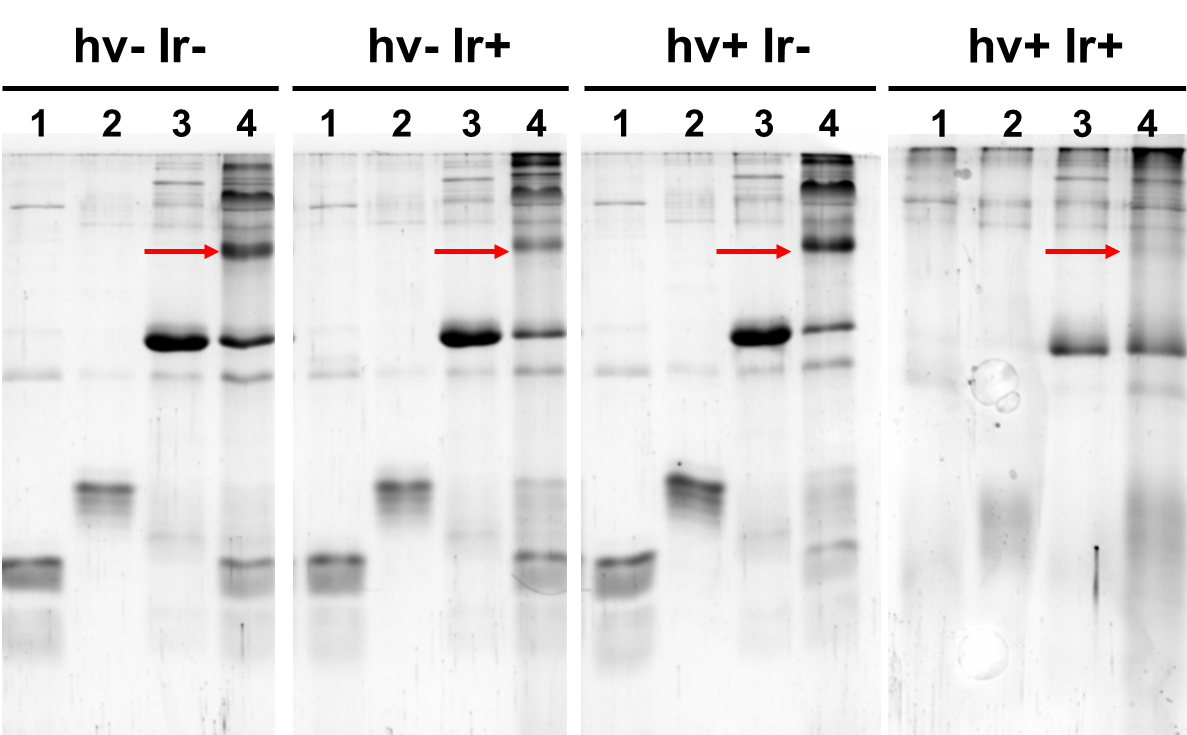
**

**Figure S36. In-gel fluorescence of coumassie blue stained SNARE proteins and their complex.** Lane 1: STX1A; lane 2: VAMP2; lane 3: SNAP-25B; lane 4: mixture of the three SNAREs. The SNARE complex is denoted by red arrow. The upper bands above the SNARE complex band indicate SNARE complex oligomers.


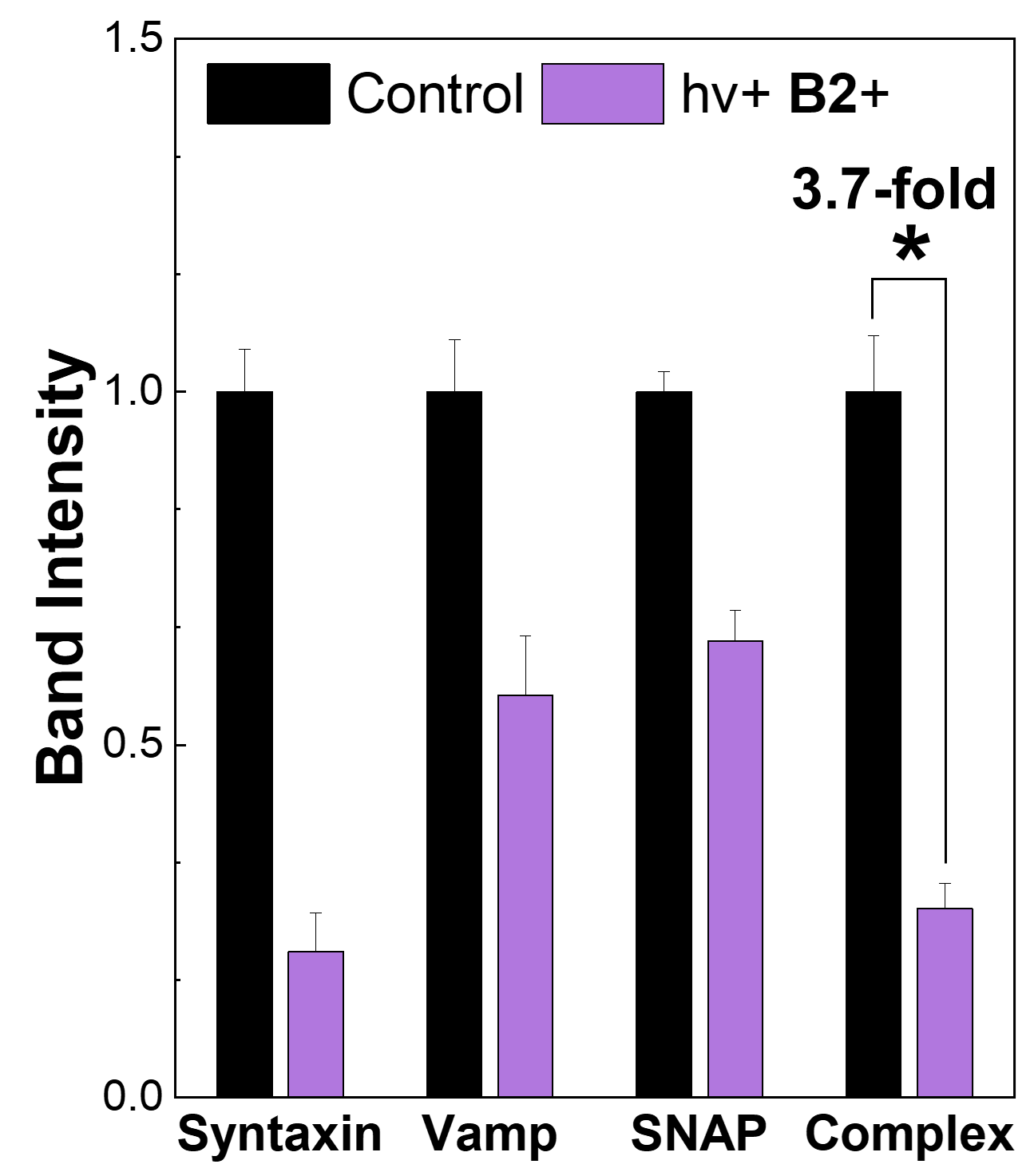


**Figure S37.** **Normalized band intensities for SNARE proteins and their complex quantifying the oxidative disruption.** The proteins were mixed and incubated at room temperature for 1 h to enable SNARE complex formation. STX: syntaxin-1A, V: VAMP2, SN: SNAP-25B. *P-value = 0.00039.


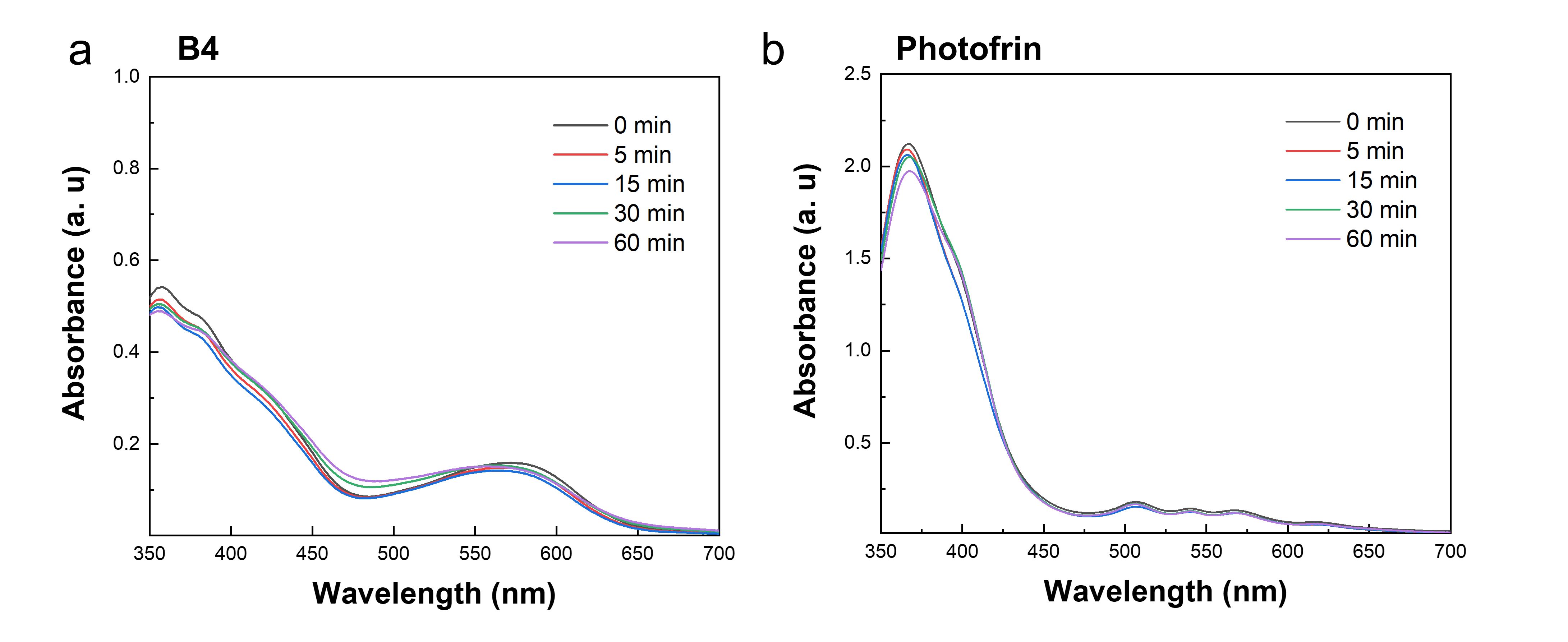


**Figure S38.** **Photostability of B4 and photofrin at different light dose.** UV-vis absorbance spectra of (a) B4 and (b) photofrin under different light dose controlled by irradiation time. 630 nm of LED was used to mimic in vivo experimental setup. Light dose: 1 min = 3 J.


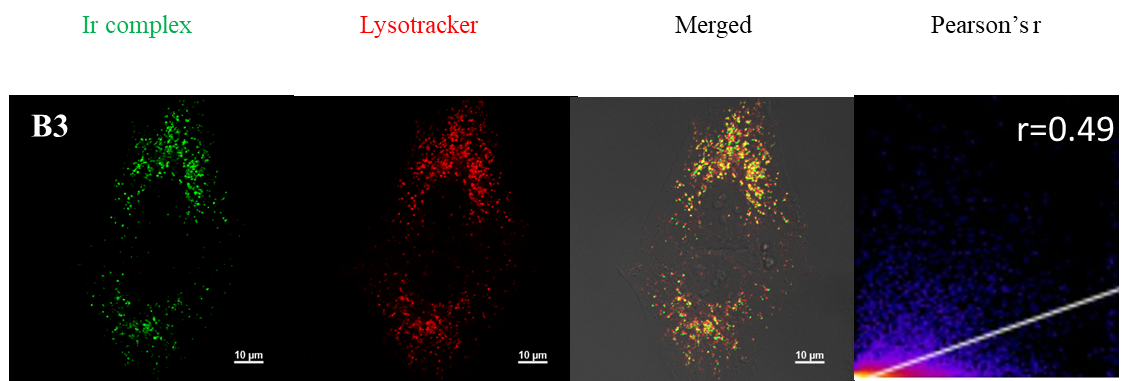


**Figure S39. Localization patterns of B3.** B3, the morpholine-free analogue of B4, were analysed with LysoTracker by confocal microscopy.

**
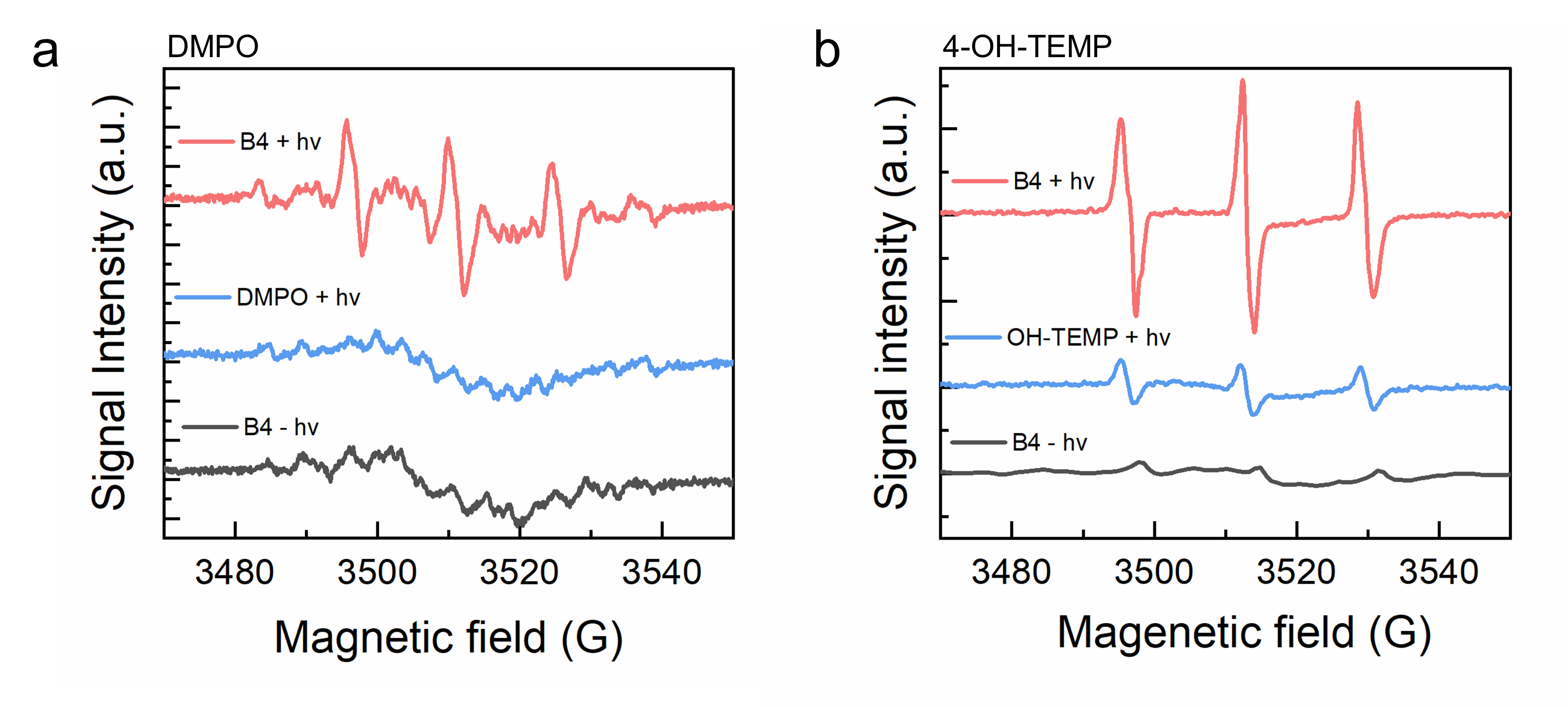
**

**Figure S40. Electron paramagnetic resonance spectra of DMPO and 4-OH-TEMP with or without B4 photosensitization.** (a) The dimerized DMPO-OH peaks detected after B4 sensitization. (b) The 4-OH-TEMPO signals observed after B4 sensitization.

**Figure S41.** **Phototoxicity and dark toxicity of B4 compared with B2.** Cytotoxicity of B2 and B4 with or without light exposure, measured by MTT assay.


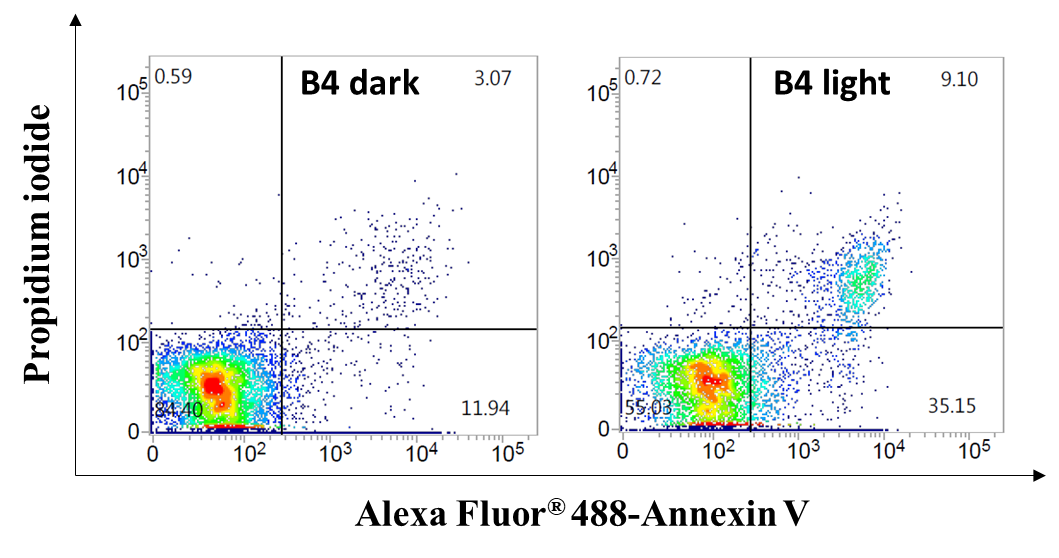


**Figure S42.** **Flow cytometry to determine apoptotic cell death by B4.** Flow cytometry results of B4 using fluorescence-activated single cell sorting with or without light irradiation.


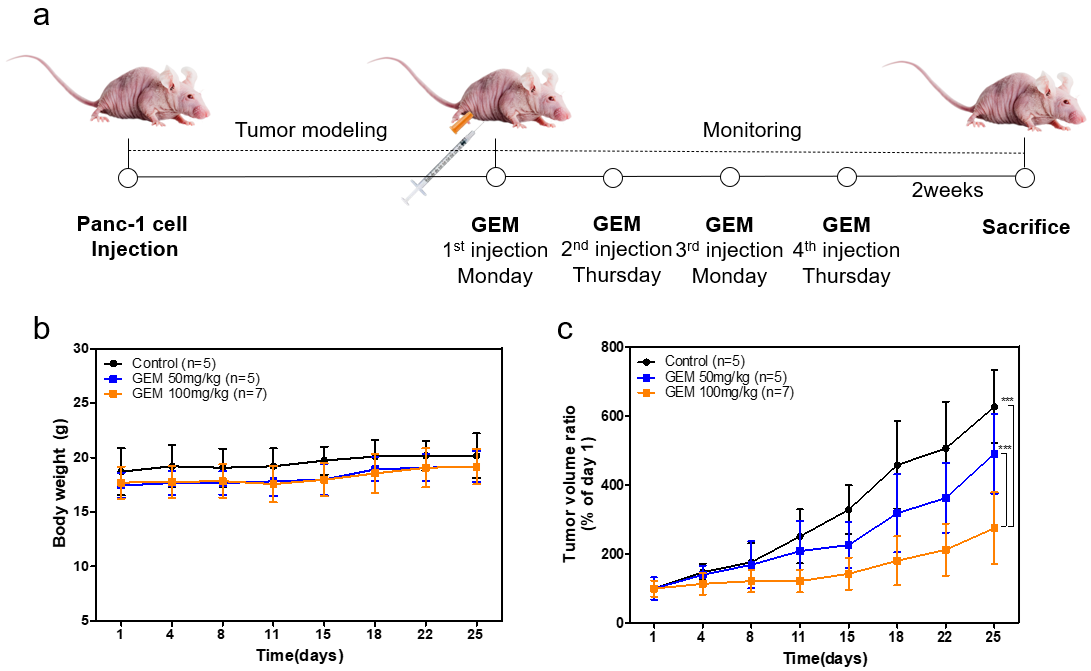


**Figure S43. Gemcitabine resistant Panc-1 tumor model.** Low dose-responsiveness of Panc-1 cell against Gemcitabine (GEM) was evaluated. (a) GEM treatment schedule. (b) Body weight of GEM treated mice over a period of 25 days. (c) Corresponding tumor growth curves for 25 days.


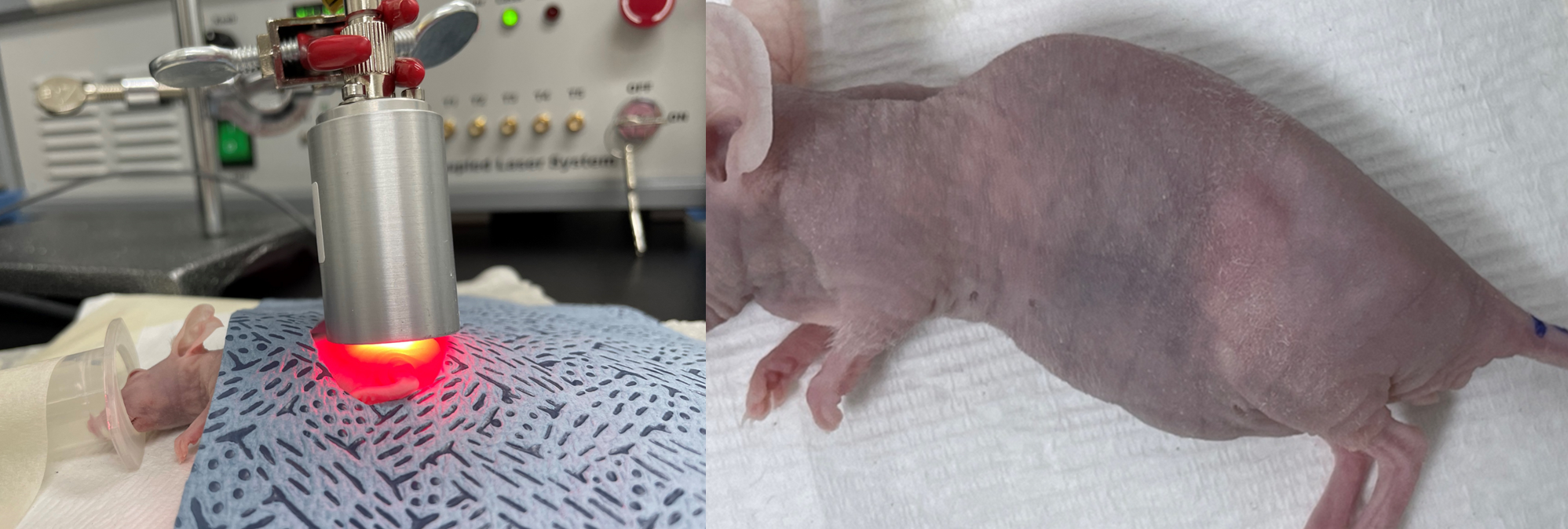


**Figure S44.** **Energy dose tolerance by 635 nm laser.** Mouse skin damage after irradiation of 635 nm laser equipped with a collimator was checked to test energy dose tolerance. No damage was observed after the irradiation of 100% intensity for 29 minutes, which corresponds with 300 J/cm^2^.

**
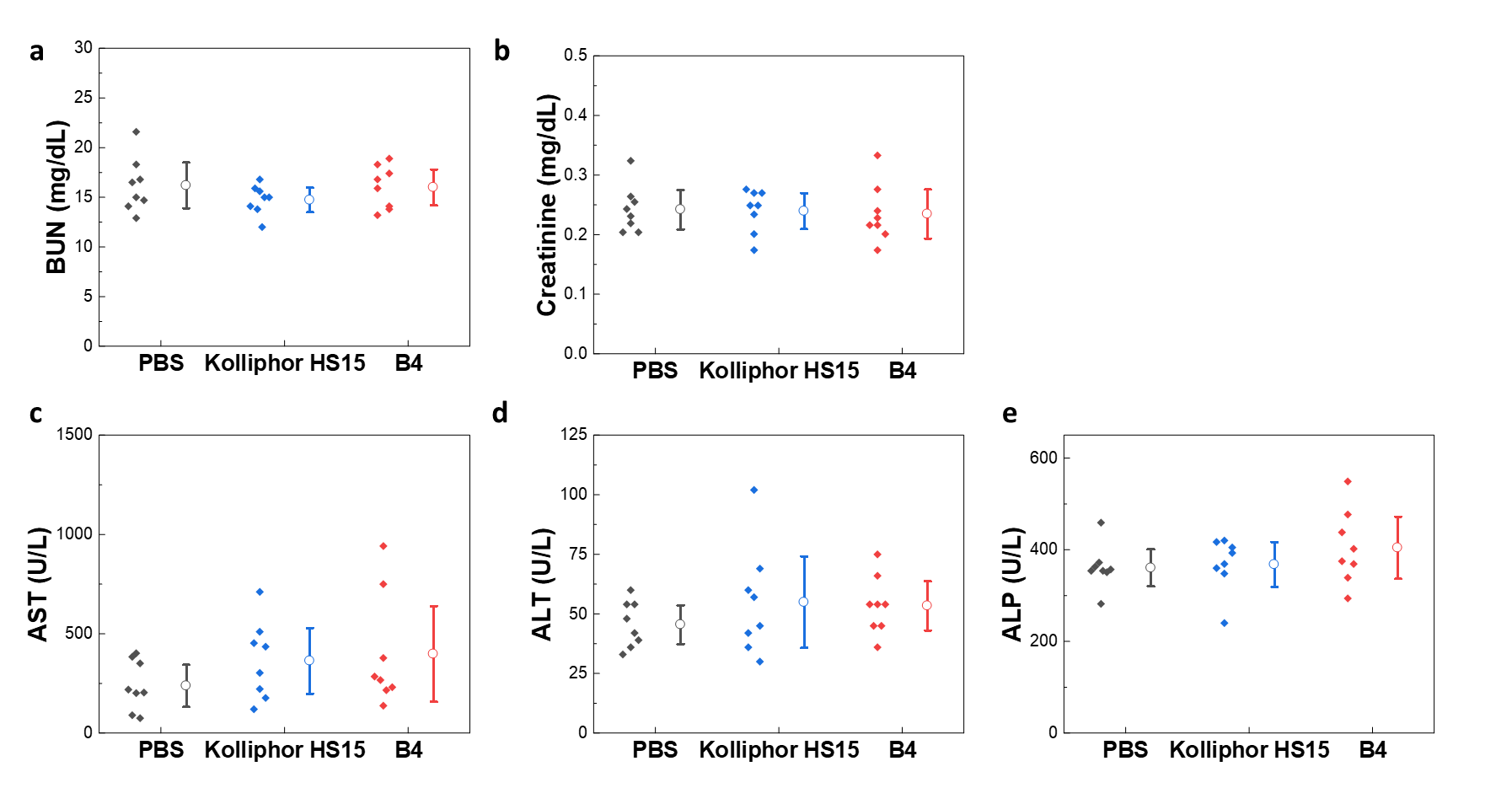
**

**Figure S45.** **Kidney and liver toxicity test.** (a,b) Kidney and (c-e) liver toxicity were evaluated by representative markers. Data points (dots) are shown with mean value (circle) and 95% confidence interval (whisker). Black: PBS, blue: 2.5% Kolliphor HS15, and red: B4 (10 mg/kg) with 2.5% Kolliphor HS15 as a solvent.

**Table S1.** Photophysical properties of Ir(III) complexes

|  | **λ_abs_^a^**  **(nm)** | **λ_em_^a^**  **(nm)** | **E_T_^b^**  **(eV)** | **Φ_PL_^c^** | **τ^d^**  **(µs)** | **k_r_^e^**  **(10^5^ s^-1^)** | **k_nr_^f^**  **(10^5^ s^-1^)** | **E_oxd_^g^**  **(V)** | **E_g_^h^**  **(eV)** | **HOMO^i^**  **(eV)** | **LUMO**  **(eV)** |
| --- | --- | --- | --- | --- | --- | --- | --- | --- | --- | --- | --- |
| **B2** | 301, 345, 358, 536 | 651 | 1.91 | 0.49 | 0.63 | 7.78 | 8.10 | 0.95 | 2.14 | -5.08 | -2.94 |
| **B4** | 304, 351, 372, 570 | 716 | 1.73 | 0.11 | 0.49 | 2.25 | 18.2 | 1.06 | 1.94 | -5.19 | -3.25 |

^a^Absorption and fluorescence spectra measured in 2×10^-5^ M CH_2_Cl_2_ solution at 300K. ^b^Triplet energy level obtained from maxima of phosphorescence spectra in frozen 2-methyl tetrahydrofuran at 77K. ^c^Phosphorescence quantum efficiency measured in degassed CH_2_Cl_2_ by the absolute method at 300K. ^d^Lifetime measured in degassed CH_2_Cl_2_ solution at 300K. ^e,f^Radiative and non-radiative rate constants were deduced by Φ_PL_ from the solution state and τ according to two equations: k_r_ = Φ_PL_/τ and k_nr_ = k_r_(1 − Φ_PL_)/Φ_PL_. ^g^Electrochemical data versus FeCp_2_^+^/FeCp_2_^0^ (FeCp_2_ = ferrocene) were collected in CH_2_Cl_2_/0.2 M TBAPF_6_ (tetrabutylammonium hexafluorophosphate) with 100 mV/s sweep rate. ^h^Optical bandgap calculated from emission spectra at 77 K. ^i^HOMO = −[4.8 − (ferrocene_oxd_) + E_oxd_] (ferrocene_oxd_ = 0.67eV).

**Table S2**. Proteins and their identified peptide sequences which contain significantly oxidized arginine or lysine residues. The oxidized amino acids are highlighted red. FC=(hv-B2-/ hv+B2+). The lower FC indicates the greater oxidation by B2.

| **Protein** | **Peptide** | **ENTREZ ID** | **uniprot ID** | **FC** |
| --- | --- | --- | --- | --- |
| **R🡪R2** |  |  |  |  |
| **SNAP23** | ITNDA**R**EDEMEENLTQVGSILGNLK | 8773 | O00161 | 0.2212 |
| **SEC22B** | **R**NLGSINTELQDVQR | 9554 | O75396 | 0 |
| **SEC22B** | VPTVS**R**PYSFIEFDTFIQK | 9554 | O75396 | 0.4567 |
| **STXBP1** | **R**EPLPSLEAVYLITPSEK | 6812 | P61764 | 0.2146 |
| **K🡪K1** |  |  |  |  |
| **STXBP3** | MLNKP**K**DK | 6814 | O00186 | 0.6263 |
| **K🡪K3** |  |  |  |  |
| **STXBP1** | V**K**EVLLDEDDDLWIALRHK | 6812 | P61764 | 0 |

**3. References**

1. Garces, F. O.; King, K. A.; Watts, R. J. Synthesis, structure, electrochemistry, and photophysics of methyl-substituted phenylpyridine ortho-metalated iridium(III) complexes *Inorg. Chem.* **1988**, *27*, 3464–3471.

2. a) Kim, H. U.; Sohn, S.; Choi, W.; Kim, M.; Ryu, S. U.; Park, T.; Jung, S.; Bejoymohandas, K. S. Substituents Engineered Deep-Red to Near-Infrared Phosphorescence from Tris-Heteroleptic Iridium(III) Complexes for Solution Processable Red-NIR Organic Light-Emitting Diodes. J. Mater. Chem. C **2018**, *6*, 10640-10658.

3. Lang, C.; Bestgen, S.; Welle, A.; Müller, R.; Roesky, P. W.; Barner-Kowollik, C. Photolithographic Encoding of Metal Complexes. *Chem. Eur. J.* **2015**, *21*, 14728–14731.

4. Wang, C.; Siriwardane, D. A.; Jiang, W.; Mudalige, T. Quantitative analysis of cholesterol oxidation products and desmosterol in parenteral liposomal pharmaceutical formulations. *Int J Pharm.* **2019**, *569*, 118576.

5. Shevchenko, A.; Tomas, H.; Havli, J.; Olsen, J. V.; Mann, M. In-gel digestion for mass spectrometric characterization of proteins and proteomes. *Nat Protoc.* **2006**, *1*, 2856–2860.
